# Photo‐Cross‐Linkable, Injectable, and Highly Adhesive GelMA‐Glycol Chitosan Hydrogels for Cartilage Repair

**DOI:** 10.1002/adhm.202302078

**Published:** 2023-10-10

**Authors:** Sattwikesh Paul, Karsten Schrobback, Phong Anh Tran, Christoph Meinert, Jordan William Davern, Angus Weekes, Travis Jacob Klein

**Affiliations:** ^1^ Centre for Biomedical Technologies Queensland University of Technology 60 Musk Ave. Kelvin Grove QLD 4059 Australia; ^2^ Department of Surgery and Radiology Faculty of Veterinary Medicine and Animal Science Bangabandhu Sheikh Mujibur Rahman Agricultural University (BSMRAU) Gazipur 1706 Bangladesh; ^3^ School of Mechanical Medical and Process Engineering Queensland University of Technology (QUT) 2 George Street Brisbane QLD 4000 Australia; ^4^ School of Biomedical Sciences Centre for Genomics and Personalised Health Translational Research Institute Queensland University of Technology (QUT) 37 Kent Street Woolloongabba QLD 4102 Australia; ^5^ Chief Executive Officer of Gelomics Pty Ltd Brisbane Queensland 4059 Australia; ^6^ ARC Training Centre for Cell and Tissue Engineering Technologies Queensland University of Technology (QUT) Brisbane QLD 4059 Australia

**Keywords:** bioadhesive, cartilage, GelMA, glycol chitosan, hydrogels, visible light

## Abstract

Hydrogels provide a promising platform for cartilage repair and regeneration. Although hydrogels have shown some efficacy, they still have shortcomings including poor mechanical properties and suboptimal integration with surrounding cartilage. Herein, hydrogels that are injectable, cytocompatible, mechanically robust, and highly adhesive to cartilage are developed. This approach uses GelMA‐glycol chitosan (GelMA‐GC) that is crosslinkable with visible light and photoinitiators (lithium acylphosphinate and tris (2,2′‐bipyridyl) dichlororuthenium (II) hexahydrate ([RuII(bpy)_3_]^2+^ and sodium persulfate (Ru/SPS)). Ru/SPS‐cross‐linked hydrogels have higher compressive and tensile modulus, and most prominently higher adhesive strength with cartilage, which also depends on inclusion of GC. Tensile and push‐out tests of the Ru/SPS‐cross‐linked GelMA‐GC hydrogels demonstrate adhesive strength of ≈100 and 46 kPa, respectively. Hydrogel precursor solutions behave in a Newtonian manner and are injectable. After injection in focal bovine cartilage defects and in situ cross‐linking, this hydrogel system remains intact and integrated with cartilage following joint manipulation ex vivo. Cells remain viable (>85%) in the hydrogel system and further show tissue regeneration potential after three weeks of in vitro culture. These preliminary results provide further motivation for future research on bioadhesive hydrogels for cartilage repair and regeneration.

## Graphical abstract

Bioadhesive hydrogels for cartilage are developed that are injectable, cytocompatible, and mechanically robust, based on gelatin methacryloyl‐glycol chitosan (GelMA‐GC) with ruthenium/sodium persulfate for photocross‐linking. Tensile and push‐out tests of the hydrogels demonstrate adhesive strength of ∼100 kPa and 46 kPa, respectively. After injection in bovine cartilage defects and in situ cross‐linking, the hydrogel remains intact and integrated with cartilage following joint manipulation *ex vivo*.

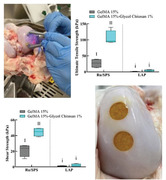

## 1 Introduction

Cartilage has little ability for self‐repair following injury. Avascularity, low cellularity, and reduced mitotic activity of chondrocytes in vivo can result in further degeneration of focal cartilage defects^[^ ^1^ ^]^ and the development of post‐traumatic osteoarthritis.^[^ ^2^ ^]^ Currently available treatment modalities, such as autologous chondrocyte implantation (ACI), grafting, arthroplasty, and microfracture, however, face major clinical challenges, for example, lack of donor availability, complicated surgical procedures, high surgery costs, and the formation of mechanically inferior fibrocartilage.^[^ ^3^ ^]^ A new generation of tissue engineering approaches is being developed to address these issues and better promote cartilage regeneration.^[^ ^4^ ^]^ In recent years, injectable hydrogels have drawn the interest of biomaterial scientists in cartilage tissue engineering^[^ ^5^ ^]^ due to ease of cell encapsulation and capacity to fill the defect using a minimally invasive approach.^[^ ^6^ ^]^ However, the mechanical properties of the hydrogels and their integration with cartilage tissue must be improved for adoption in cartilage repair and regeneration.

Hydrogel mechanical properties play a crucial roles in both load‐bearing during daily activities and fostering the development and maturation of engineered tissues. Although synthetic polymers can be used to generate strong hydrogels, they often lack cell‐specific bioactivities like cell adhesion and biodegradation sites,^[^ ^7^ ^]^ necessitating the incorporation of bioactive molecules,^[^ ^8^ ^]^ or alternatively selecting natural polymers. Among the natural polymers, chitosan, a deacetylated derivative of chitin, is promising for cartilage repair due to its resemblance to extracellular matrix glycosaminoglycans (GAG),^[^ ^9^ ^]^ and adhesive properties governed by ionic interactions with tissues or mucus layers.^[^ ^10^ ^]^ However, chitosan is poorly soluble in water and necessitates an acidic pH for effective polymer dissolution, raising toxicity concerns for protein and cell delivery applications.^[^ ^11^ ^]^ On the other hand, glycol chitosan (GC), a water‐soluble chitosan derivative, preserves the desirable properties of chitosan while enhancing cell compatibility,^[^ ^12^ ^]^ and has been implemented for cartilage tissue engineering.^[^ ^13^ ^]^ Whereas chitosan and derivatives produce brittle hydrogels,^[^ ^14^ ^]^ combining them with other natural polymers can enhance the mechanical properties.^[^ ^14^, ^15^ ^]^ An ideal candidate for this second polymer is gelatin methacryloyl (GelMA), a collagen‐based, photo‐cross‐linkable material with adaptable mechanical properties and excellent biocompatibility.^[^ ^16^ ^]^

Integration between the biomaterial and native tissue is equally important as the hydrogel mechanical properties, providing both mechanical stability and initiating cellular crosstalk and matrix accumulation at the interface for long‐term adhesive strength. Bioadhesive hydrogels may provide solutions to the challenges associated with hydrogel–cartilage integration. Bioadhesives act through a number of mechanisms to bind tissues together.^[^ ^17^ ^]^ In addition to providing physical support, bioadhesives can convey bioactive cues and cells^[^ ^18^ ^]^ for use in tissue repair and regenerative medicine. ^[^ ^19^ ^]^ Commonly used bioadhesives include cyanoacrylates, aldehydes, fibrin glue, and polyethylene glycol (PEG) derivatives.^[^ ^20^ ^]^ Cyanoacrylates have excellent hemostasis, quick and strong adhesion, and bacteriostatic properties;^[^ ^17^, ^21^ ^]^ however, their by‐products may be cytotoxic and result in foreign‐body reactions and tissue necrosis.^[^ ^22^ ^]^ Although aldehyde‐based bioadhesives have bonding strength similar to cyanoacrylates, formaldehyde by‐products may cause cytotoxicity, mutagenicity, and carcinogenicity.^[^ ^23^ ^]^ Fibrin glue is frequently used in surgery,^[^ ^20^, ^24^ ^]^ but poor bond strengths (≈0.001–0.027 MPa)^[^ ^25^ ^]^ relative to synthetic tissue adhesives (≈0.6–3 MPa),^[^ ^26^ ^]^ and rapid degradation restrict its application. ^[^ ^27^ ^]^ PEG‐based adhesives have high swelling ratios (SR),^[^ ^17^, ^28^ ^]^ which may result in compromised adhesion^[^ ^29^ ^]^ and tissue compression. ^[^ ^30^ ^]^ A chondroitin sulfate (CS)‐based PEG diacrylate (PEGDA) hydrogel has shown some promising results in cartilage regeneration and adhesive strength in vitro and in vivo,^[^ ^31^ ^]^ however, this required an additional adhesive layer between the scaffold and cartilage. A double‐network of PEG dimethacrylate and alginate reinforced with nano‐fibrillar cellulose developed considerable adhesive strength, ≈130 kPa with articular cartilage;^[^ ^32^ ^]^ however, this adhesive was cured for a long (30 min) period with UV, and its durability in a joint context was not assessed. Gelatin‐based bioadhesives have also been reported,^[^ ^33^ ^]^ and GelMA has been used as a copolymer for wound adhesives and lung sealants with high biocompatibility.^[^ ^34^ ^]^ Recently, microbial transglutaminase was added to GelMA for testing the adhesion to human cartilage in vitro;^[^ ^35^ ^]^ however, the adhesive strength remained within the range of fibrin glue.^[^ ^25^ ^]^ The adhesiveness of GelMA may be improved by changing the cross‐linking conditions. GelMA is cross‐linkable with visible light and photoinitiators (lithium acylphosphinate (LAP) and tris (2,2′‐bipyridyl) dichlororuthenium (II) hexahydrate ([RuII(bpy)_3_]^2+^ and sodium persulfate (Ru/SPS)), with Ru/SPS offering the potential benefit of di‐tyrosine bond formation between GelMA and native tissue.^[^ ^36^ ^]^

Achieving a combination of desirable material properties and adhesive strength as a one‐pot formulation for cartilage tissue engineering remains an unresolved challenge that must be addressed. Therefore, the aim of this study was to develop a natural polymer‐based injectable and bioadhesive visible light cross‐linked hydrogel with suitable mechanical and adhesive properties for cartilage repair. We hypothesized that a new combination of GelMA and GC would act through multiple mechanisms to deliver these properties. We prepared and evaluated the GelMA‐GC hydrogels in a range of in vitro and ex vivo experiments in this study.

## 2 Results and Discussion

### 2.1 Addition of GC Increases Compressive Modulus

**Figure 1:**
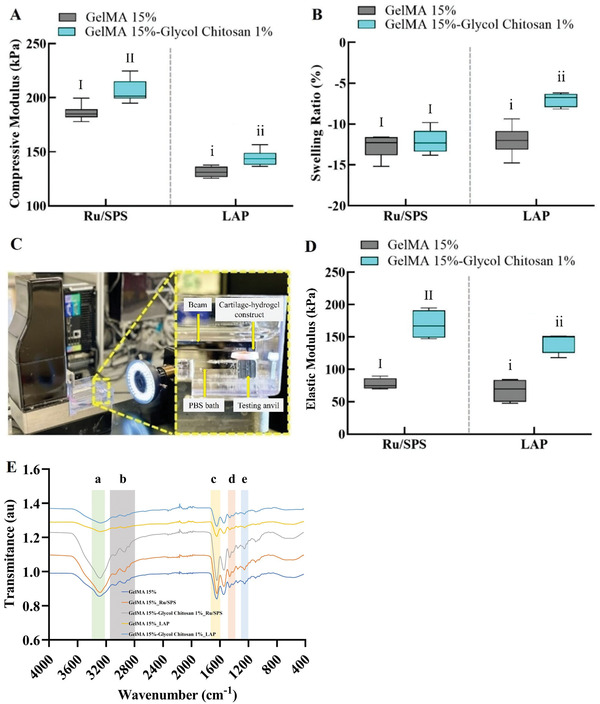
Compressive modulus, swelling ratio, elastic modulus, and FTIR spectra of GelMA and GelMA‐GC hydrogels. 15% (w v^−1^) GelMA alone or blended with 1% (w v^−1^) GC was cross‐linked with Ru/SPS or LAP photoinitiators at 405 nm, respectively. A) Compressive moduli of hydrogels were measured 24 h after polymerization using an Instron 5567 tester with a nonporous indenter and a 500 N load cell. B) The swelling ratio was determined by weighing hydrogels directly after polymerization and after 24 h of incubation in PBS (pH = 7.4); sample size *n* = 6 per group. C) Microindentation test performed at 37 °C in Cellscale microtester. D) Elastic modulus calculated from the microindentation test by Cellscale microtester at 37 °C in PBS (pH = 7.4) bath; sample size *n* = 4 per group. Groups that do not share a common Roman numeral are statistically different (*p* < 0.05). The comparison was conducted just inside each cross‐linker group due to the differences in the cross‐linking conditions and indicated by capital and lowercase numerals. GelMA (15%, w v^−1^) hydrogels were considered as control in each group. E) FTIR‐ATR spectra of non‐crosslinked GelMA, as well as GelMA and GelMA‐GC hydrogels with highlighted regions showing a) O─H and N─H stretching vibrations, b) C─H stretching vibrations, c) C═O stretching, amide I, d) N─H bending coupled to C─H stretching, amide II, and e) C─N stretching and N─H bending, amide III.

To determine the effects of GC on the mechanical properties of GelMA‐GC hydrogels, compression test and swelling analysis were conducted with two different photo‐cross‐linking systems, Ru/SPS and LAP, respectively. GC significantly increased the CM of hydrogels in both photoinitiator groups (**Figure** **1**A). GelMA‐GC hydrogels cross‐linked by Ru/SPS and LAP revealed higher CM (205.8 ± 10.8 and 144.2 ± 7.3 kPa, respectively) compared to GelMA hydrogels (186.1 ± 7.2 and 131.3 ± 4.9 kPa, respectively) in their specific groups. The SR did not change with the addition of GC in the Ru/SPS group with GelMA; however, in the LAP group, the GelMA hydrogels showed a significantly lower SR compared to GelMA‐GC hydrogels (Figure 1B).

The mechanical characteristics of cell‐free Ru/SPS and LAP cross‐linked GelMA and GelMA‐GC hydrogels in the central defect model of bovine articular cartilage constructs were examined using a nondestructive microindentation test after overnight incubation at 37 °C (Figure 1C). The elastic modulus (*E*) was calculated using the Hertz contact model for a spherical indenter. ^[^ ^37^ ^]^ GC significantly enhanced the *E* of hydrogels compared to GelMA hydrogels on both photoinitiator groups (Figure 1D). The Ru/SPS cross‐linked GelMA‐GC hydrogels showed an increase in *E* (168.9 ± 22.1 kPa) compared to GelMA hydrogels (77.2 ± 8.6 kPa). Moreover, LAP cross‐linked GelMA‐GC hydrogels showed a significantly higher *E* (142.2 ± 16.2 kPa) compared to GelMA hydrogels (67.6 ± 17.9 kPa) (Figure 1D).

We prepared hydrogels utilizing two visible light photoinitiators, Ru/SPS and LAP, which can influence the hydrogel mechanical properties.^[^ ^38^ ^]^ Photoinitiators are classified into type I and type II, with type I consisting of only one component and type II requiring two components, a photoinitiator and co‐initiator.^[^ ^39^ ^]^ LAP is a type I photoinitiator with low molar absorptivity in a narrow visible light range (*ε* ≈ 30 M^−1^ cm^−1^ at 405 nm)^[^ ^40^ ^]^ and cross‐linking is accomplished solely through radical polymerization.^[^ ^41^ ^]^ However, Ru/SPS is a type II photoinitiator with a higher molar extension coefficient in the visible light range (*ε* ≈ 14 600 M^−1^ cm^−1^ at 450 nm) and cross‐linked the polymers in two ways: firstly by radical polymerization and secondly by di‐tyrosine bond formation in the available protein groups,^[^ ^41^, ^42^ ^]^ which may have increased the CM of hydrogels in that group. When exposed to visible light, photoexcited Ru^2+^ oxidizes into Ru^3+^ by giving electrons to SPS, and SPS dissociates into sulfate anions and sulfate radicals.^[^ ^43^ ^]^ As a result, the radicals propagate through the methacryloyl groups, forming cross‐links in GelMA.^[^ ^41^, ^42^ ^]^ Moreover, photoexcited Ru^3+^ is responsible for the formation of cross‐links with the phenol moieties, such as tyrosine, present in the GelMA backbone.^[^ ^41^, ^42^, ^43^ ^]^ These oxidized tyrosine groups are transformed into tyrosyl radicals, which are then neutralized by the formation of di‐tyrosine bonds with other adjacent tyrosine groups.^[^ ^36^ ^]^ The Fourier‐transform infrared (FTIR) spectrum of hydrogels containing GelMA reveals characteristic peaks of the gelatin polymer (Figure 1E). The absorption band at ≈3346 cm^−1^ is attributed to the stretching vibrations of O─H and N─H bonds. Peaks within the 2800–3100 cm^−1^ region correspond to the stretching vibrations of C─H groups.^[^ ^44^ ^]^ Noteworthy peaks at 1651, 1544, and 1253 cm^−1^ signify C═O stretching, N─H bending coupled with C─H stretching, and C─N stretching with N─H bending, respectively.^[^ ^45^ ^]^ The prominent peak centered at 1651 cm^−1^ signifies the conversion of C═C double bonds of methacrylate or methacrylamide groups.^[^ ^46^ ^]^ The FTIR spectra provide evidence that the reactive groups within the hydrogels undergo cross‐linking facilitated by visible light photoinitiators.

Inclusion of GC significantly increased the CM of hydrogels cross‐linked by both photoinitiators in two mechanical testing setups (Figure 1A,D). The addition of GC might have enhanced the CM by forming H‐bonds with the GelMA network.^[^ ^47^ ^]^ In the FTIR spectra of polymers, distinct intermolecular forces such as hydrogen bonds, ions, and charge transfers often manifest in the spectral range of 1100–1200 cm^−1^.^[^ ^48^ ^]^ Notably, within this range, a more pronounced peak emerges in the FTIR spectra of Ru/SPS cross‐linked hydrogels when compared to LAP cross‐linked hydrogels (Figure 1E). Furthermore, GC is a polycationic polymer, and we hypothesized that it might have formed ionic interactions with the sulfate anions generated during photo‐polymerization and further strengthened CM; however, further studies are needed to test this hypothesis. The elevated solid content observed in GelMA‐GC hydrogels in comparison to GelMA emerges as a plausible additional factor contributing to the enhanced mechanical properties of hydrogels. Consequently, an assessment of the impact of increased solid content on hydrogel mechanical characteristics was undertaken, employing GelMA at a concentration of 16% (w v^−1^) (Figure S1,). The CM of 16% (w v^−1^) GelMA was similar to those of the GelMA‐GC hydrogels within both photoinitiator groups (Figure S1, Supporting Information). In addition, within both photoinitiator groups, the hydrogels formulated with 16% (w v^−1^) GelMA displayed an elastic modulus comparable to that of GelMA‐GC hydrogels, thereby underscoring the influence of increased polymer solid content on mechanical properties (Figure S1, Supporting Information).

This study represents promising mechanical properties with the GelMA‐GC combination at a ratio of 15% (w v^−1^) GelMA and 1% (w v^−1^) GC. This specific combination was chosen following extensive experimental evaluations, involving acid soluble chitosan concentrations of 0.5%, 1%, and 1.5% (w v^−1^), alongside varying concentrations of GelMA (10%, 15%, and 20%, w v^−1^). Among these, the 1% (w v^−1^) chitosan group combined with 15% (w v^−1^) GelMA exhibited promising outcomes in relation to hydrogel formulation and mechanical properties (data not shown). Subsequent investigations were carried out to validate this combination by exploring varying concentrations of GelMA (10% and 20%, w v^−1^) while maintaining a constant 1% (w v^−1^) GC content (Figure S1, Supporting Information). Moreover, considering the potential concerns associated with injectability and biodegradability with higher polymer concentrations, the subsequent investigations were undertaken using the combination of 15% (w v^−1^) GelMA and 1% (w v^−1^) GC.

The CM of native articular cartilage is ≈0.5 MPa and may vary depending on the location in the body and age.^[^ ^49^ ^]^ Although the CM of Ru/SPS cross‐linked GelMA‐GC hydrogels at day 1 of culture are lower than that of native cartilage, this is promising for cartilage regeneration because the maintenance of chondrocyte phenotype, re‐differentiation, GAG, and collagen II accumulation can be achieved in a hydrogel construct with stiffness of around 30 kPa. ^[^ ^50^ ^]^ We hypothesize that after application in vivo, this hydrogel system will facilitate the growth and maturation of regenerated tissue structures, resulting in increased mechanical stability of the hydrogel system.

### 2.2 Addition of GC Increases Adhesive Properties

**Figure 2:**
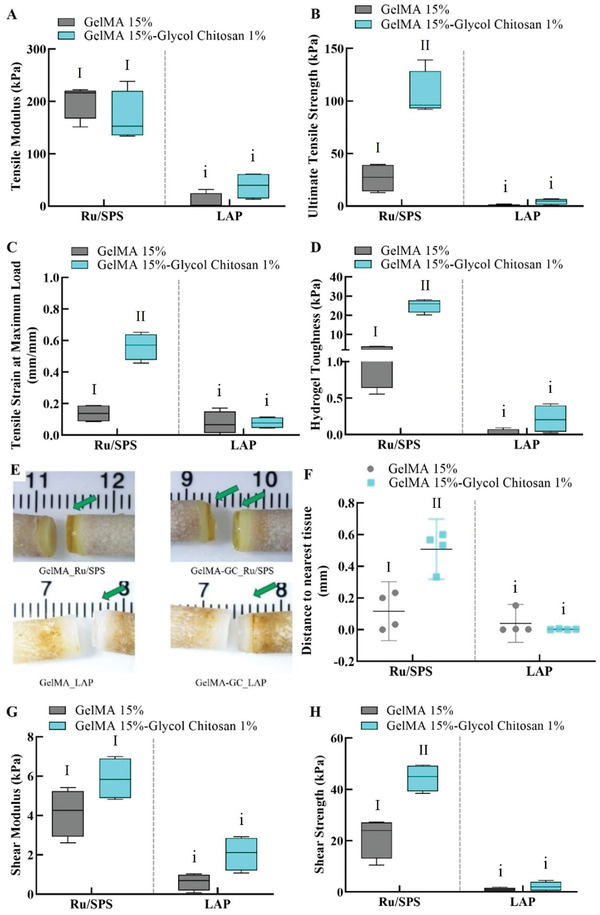
Effects of glycol chitosan on adhesive properties of GelMA and GelMA‐GC hydrogels. 15% (w v^−1^) GelMA alone or blended with 1% (w v^−1^) GC hydrogels were cross‐linked with Ru/SPS and LAP photoinitiators in between the cartilage surfaces of bovine osteochondral constructs. A) Tensile modulus, B) ultimate tensile strength, C) tensile strength at maximum load, and D) hydrogel toughness was measured after polymerization and after 1 hour incubation with PBS (pH = 7.4). E) Images of the osteochondral constructs and hydrogels after adhesion tests; green arrows indicate hydrogel–cartilage integration. F) Length of hydrogel to nearest cartilage tissue was measured after adhesion tests (0 mm indicates no hydrogel on the nearest tissue). G) Shear modulus and H) shear strength measured in push‐out test after overnight incubation in PBS at 37 °C. Groups that do not share a common Roman numeral are statistically different (*p* < 0.05). The comparison was conducted just inside each cross‐linker group, as indicated by capital and lowercase numerals. GelMA (15%, w v^−1^) hydrogels were considered as control in each group. Sample size *n* = 4 per group; error bars: Mean ± SD.

Adhesive properties of the GelMA and GelMA‐GC hydrogels were assessed by uniaxial tensile and push‐out tests using bovine osteochondral constructs to investigate their adhesiveness to the native cartilage tissue. Addition of GC to GelMA‐based hydrogels had a substantial impact on hydrogel ultimate tensile strength (UTS), tensile strain at maximum load (TSML), and toughness in Ru/SPS cross‐linked hydrogels. In contrast, the influence of GC on any of the adhesive characteristics was not significant in the LAP cross‐linked group (**Figure** **2**A–C). The Ru/SPS cross‐linked GelMA‐GC hydrogels had a comparable tensile modulus (TM) (169.3 ± 47.9 kPa) to GelMA hydrogels (201.5 ± 33.6 kPa) (Figure 2A). GC resulted in a significant increase of UTS with GelMA in the Ru/SPS group compared to GelMA controls (Figure 2B). Moreover, Ru/SPS cross‐linked GelMA‐GC hydrogels had significantly higher TSML (0.56 ± 0.08 mm mm^−1^) than GelMA hydrogels (0.14 ± 0.06 mm mm^−1^) (Figure 2C). Furthermore, GC significantly increased hydrogel toughness of GelMA in the Ru/SPS group compared to controls (Figure 2D).

The adhesive and cohesive failure of hydrogels in the osteochondral construct was determined based on the adherent hydrogel length (mm) from the nearest cartilage tissue observed in stereomicroscopic images obtained following the uniaxial tensile test. The Ru/SPS cross‐linked GelMA‐GC hydrogels showed failure in the hydrogel itself (cohesive failure); however, in the GelMA hydrogels, failure occurred most frequently from the hydrogel–cartilage interface (adhesive failure) (Figure 2E). The addition of GC to GelMA significantly increased adherent hydrogel length (0.47 ± 0.1 mm) over GelMA (0.05 ± 0.1 mm) in the Ru/SPS group (Figure 2F). In contrast, GelMA and GelMA‐GC hydrogels from the LAP cross‐linked group exhibited comparable adhesive failure (Figure 2E,F).

Addition of GC showed an increasing trend of shear modulus in both photoinitiators group on day 1; however, values were similar to their respective controls (Figure 2G). Cell‐free Ru/SPS cross‐linked GelMA‐GC hydrogels showed significantly higher shear strength (44.5 ± 5.3 kPa) than GelMA hydrogels (21.4 ± 7.9 kPa) on day 1 (Figure 2H). On the other hand, addition of GC in LAP cross‐linked GelMA hydrogels did not affect shear strength of GelMA hydrogels (Figure 2H).

Robust integration between engineered and native tissue is crucial for enhanced matrix accumulation at the interface, resulting in increased bonding strength. Improved bonding strength at the cartilage–hydrogel interface has been attributed to collagen neo‐synthesis and cell invasion.^[^ ^51^ ^]^ However, the antiadhesive properties of GAGs^[^ ^52^ ^]^ make the lateral integration of implant material more challenging. Various strategies have been investigated to improve the mechanical stability between an implant and host articular cartilage, including enzymatic pretreatment,^[^ ^53^ ^]^ utilizing chemotactic agents,^[^ ^54^ ^]^ or creating an adhesive interface between scaffolds and host tissue.^[^ ^31^, ^55^ ^]^ However, these approaches are time‐consuming and susceptible to host tissue damage or improper integration if not performed properly, which can result in a weaker implant–cartilage integration. After an injury, the joint remains in an inflammatory state, with increased levels of pro‐inflammatory cytokines that mediate cartilage matrix breakdown.^[^ ^56^ ^]^ One option for overcoming the integration difficulty is to utilize biomaterials with adhesive features that will cover the inflammatory joint surface and secure a robust integration of implant or biomaterial with natural cartilage at time zero. Fibrin‐based surgical sealants (e.g., Tisseel) have been used but have shown unsatisfactory outcomes in a rabbit model.^[^ ^57^ ^]^ In cartilage defects following microfracture, a chondroitin sulfate adhesive was employed in conjunction with a polyethelene glycol/hyaluronic acid hydrogel;^[^ ^58^ ^]^ nonetheless, delamination was detected in 25% of patients. Chitosan‐based bioadhesive glue such as BST CarGel and JointRep show encouraging results,^[^ ^59^ ^]^ but are only useful in conjunction with microfracture surgery. In contrast, without cartilage pretreatment or surgical intervention like micro‐fracturing, we measured adhesive strength of ≈100 kPa in tensile tests for Ru/SPS cross‐linked GelMA‐GC (Figure 2B). Moreover, the shear strength of cell‐free Ru/SPS cross‐linked GelMA‐GC hydrogels on day 1 was significantly higher (≈45 kPa) than that of GelMA controls (Figure 2H). Furthermore, this shear strength is higher than other GelMA hydrogels and previously reported bioadhesive hydrogels such as factor XIII cross‐linked hyaluronan hydrogels (adhesive strength ≈ 6 kPa),^[^ ^60^ ^]^ dual‐functionalized tyramine and methacryloyl gelatin (GelMA‐Tyr) hydrogel bio‐ink (adhesive strength ≈ 13 kPa),^[^ ^61^ ^]^ microbial transglutaminase treated GelMA hydrogels (adhesive strength ≈ 6 kPa),^[^ ^35^ ^]^ chondroitinase ABC (cABC) treated HA‐Tyr bioadhesive hydrogels (adhesive strength ≈ 35 kPa),^[^ ^62^ ^]^ and fibrin‐based adhesives (adhesive strength ≈ 1–27 kPa).^[^ ^63^ ^]^ This suggests that the addition of polycationic GC and the use of Ru/SPS cross‐linking improved the adhesive strength of the hydrogel. This may occur through electrostatic interactions between GC and biological surfaces containing anionic GAG, Ru/SPS‐mediated di‐tyrosine bonding between GelMA and proteins at the defect site, or other mechanisms. While we did observe a discernible impact of elevated polymer solid content on the mechanical properties of the hydrogel within both photoinitiator groups (Figure S1, Supporting Information), it did not yield improvements in the tensile and shear strength (Figure S2B,H, Supporting Information). This observation suggests a potential synergistic effect attributed to the combination of GC and Ru/SPS cross‐linking.

In this study, the adhesive strength of hydrogels was evaluated using native bovine cartilage. When evaluating the adhesion strength of a bioadhesive for cartilage repair, it is crucial to execute the evaluation with adequate hydration, as cartilage is surrounded by synovial fluid. A notable advantage of this study is the consistent maintenance of adequately hydrated conditions in both tests. In the tensile test, the constructs were incubated at 37 °C for 1 h and maintained in a PBS until prior to placement in the mechanical tester. Furthermore, the hydrogel–cartilage junction was covered with a silicone tube to prevent the drying out of both the construct and hydrogels. The tube was removed just prior to the commencement of the test, which lasted ≈2 min; it is presumed that the sample remained adequately hydrated throughout this period. The push‐out test was executed following an overnight incubation of the cartilage–hydrogel constructs at 37 °C, and the entire procedure was accomplished by submerging the construct in PBS at 37 °C.

Bioadhesive properties are defined by the cohesive strength of the material and the adhesive strength between the material and adherent tissue.^[^ ^64^ ^]^ It is critical to find the right balance between the adhesive and cohesive strengths of a bioadhesive to ensure optimal bonding performance.^[^ ^65^ ^]^ The adhesive or cohesive failure of hydrogels was investigated in this study using stereomicroscopic images of osteochondral constructs following a uniaxial tensile test (Figure 2E). A quantitative analysis of the length of adherent hydrogels from the nearest cartilage tissue end of the osteochondral constructs showed a promising adhesive bond in Ru/SPS cross‐linked GelMA‐GC hydrogel, further validating our hypothesis on Ru/SPS and GC‐mediated cross‐linking (Figure 2E,F). However, adhesive failure was detected in LAP cross‐linked GelMA‐GC hydrogels, which may be attributable to the absence of di‐tyrosine bonds where only GC‐mediated electrostatic interactions were insufficient to maintain cartilage‐hydrogel integration (Figure 2E,F).

Human cartilage tensile strength ranges between 8.1 and 40 MPa, depending on location and age.^[^ ^66^ ^]^ It is difficult to recommend an appropriate adhesive strength for cartilage tissue adhesive due to the variability of lesions, treatment approaches and individual circumstances. The UTS of meniscus tissue in the circumferential direction is estimated to be between 12 and 19 MPa.^[^ ^67^ ^]^ However, an adhesive strength of a tissue adhesive within a range of 50–100 kPa has been described as sufficient for meniscus repair.^[^ ^67a^ ^]^ Although no optimal bonding strength for a cartilage bioadhesive has been clinically described, achieving an adhesive strength within the range, comparable to or higher than that of native cartilage tensile strength is the most desirable. To our knowledge the ideal adhesive strength for a cartilage bioadhesive has not been clinically described, although a CS adhesive with poly(ethylene glycol) diacrylate (PEGDA) hydrogels showed promising cartilage integration and repair in a goat model with tensile and shear strengths of 45 and 46 kPa, respectively.^[^ ^31^ ^]^ Moreover, a hybrid photo‐cross‐linkable (HPC) hydrogel with uniaxial tensile and horizontal shear strengths of 16 and 29 kPa was effectively administered arthroscopically in a swine model.^[^ ^68^ ^]^ From all of these observations, we hypothesize that the Ru/SPS cross‐linked GelMA‐GC hydrogel, with its promising tensile strength (≈100 kPa) and shear strength (≈45 kPa) will ensure secure integration after in vivo application. However, rigorous in vivo studies are required to confirm our hypothesis.

### 2.3 GelMA‐GC Hydrogels Cross‐Linked by Ru/SPS Were Durable in Bovine Cartilage Defects

**Figure 3:**
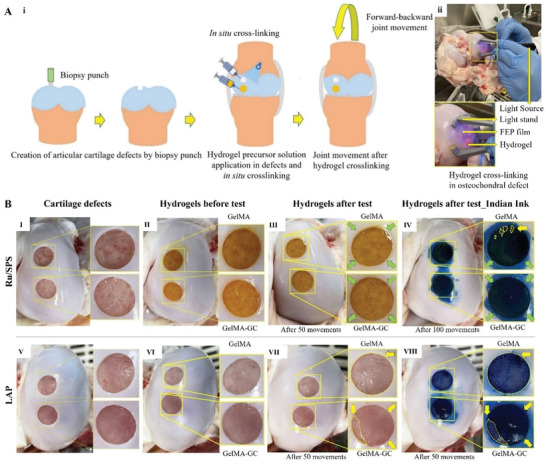
Ex vivo adhesion test of in situ cross‐linked hydrogels in a bovine stifle joint. A) Schematic representation of ex vivo joint movement study after application and in situ cross‐linking of 15% (w v^−1^) GelMA alone or blended with 1% (w v^−1^) GC hydrogels with Ru/SPS or LAP photoinitiator in knee osteochondral defects. B) Osteochondral defects creation by biopsy punch (diameter: 10 mm), hydrogel precursor solution application and cross‐linking (Ru/SPS: 5 min; LAP: 3 min at 405 nm), hydrogels in osteochondral defects after manual joint movement, India Ink staining of Ru/SPS cross‐linked (I–IV) and LAP cross‐linked (V–VIII) GelMA and GelMA‐GC hydrogels. Integration of hydrogels with native cartilage after joint movement study (good integration marked as green arrows; weak integration marked as yellow arrows), areas with dislodged hydrogels are marked with yellow contour lines. Hydrogels were stained with India Ink for visualization purposes.

To test the durability of GelMA‐GC hydrogels under joint conditions, a bovine ex vivo joint study was conducted. The Ru/SPS cross‐linked hydrogels with or without GC demonstrated uniform surface morphology and promising adhesion to cartilage defects after joint loading for 50 cycles (marked as green arrows) (**Figure** **3**B III; Video S1, Supporting Information). In contrast, in the LAP cross‐linked group, GelMA was completely dislodged from the cartilage defect site after only six loading cycles, which is marked with yellow contour lines (Figure 3B VII; Video S2, Supporting Information). Moreover, GelMA‐GC hydrogel of the same photoinitiator group broke apart and lost the integration with cartilage after loading cycle for 50 times, which is also marked as yellow dotted area (Figure 3B VII). After India Ink staining and 100 times joint cycles, points of weak hydrogel–cartilage integration (marked as yellow arrow) were revealed in GelMA hydrogels of the Ru/SPS group; while GelMA‐GC hydrogel exhibited a robust integration with native cartilage (green arrows; Figure 3BIV). Moreover, part of Ru/SPS cross‐linked GelMA hydrogels were broken and exposed the defect site (yellow contour lines; Figure 3BIV). The India Ink stained the total cartilage defect area as the LAP cross‐linked GelMA hydrogel was completely dislodged from the defect (yellow contour lines; Figure 3B VIII). Moreover, the ink stained the exposed part of the cartilage defect and underneath areas of LAP cross‐linked GelMA‐GC hydrogel as cartilage‐hydrogel integration was lost (marked as yellow contour lines and arrows respectively; Figure 3B VIII). The Ru/SPS cross‐linked GelMA and GelMA‐GC hydrogels remained within the defect after applying gentle pressure with the fingertip (20 times), while the LAP cross‐linked GelMA‐GC hydrogel completely dislodged from the defect after 1–2 pushes with the fingertip, indicating very weak adhesion with the native cartilage (Video S3, Supporting Information).

We hypothesized that the adhesive strength of Ru/SPS cross‐linked GelMA‐GC hydrogels was sufficient to guarantee secure fixation of hydrogels with damaged cartilage based on the tensile and push‐out test outcomes (Figure 2). In order to visualize the adhesive and cohesive strength of Ru/SPS cross‐linked GelMA‐GC hydrogels in a joint context, an ex vivo joint study was conducted. The matrix of tough adhesive hydrogel is highly resistant to crack propagation and adhere firmly to various substrates.^[^ ^69^ ^]^ We hypothesized that our Ru/SPS cross‐linked GelMA‐GC hydrogels, with toughness around 25 kPa (Figure 2D), would remain stable during joint movement. The joint study revealed that Ru/SPS cross‐linked GelMA‐GC hydrogels were strongly integrated with cartilage, as evidenced by the lack of any gaps between the hydrogel and cartilage after staining with India Ink (Figure 3B IV). The hydrogels were so well integrated with the cartilage that it was difficult to remove them from the defects with fingertip pressure (20 times) (Video S3, Supporting Information). However, LAP cross‐linked hydrogels failed to maintain robust hydrogel–cartilage integration, further validating our hypothesis of LAP‐mediated cross‐linking in this study (Figure 3B VII,VIII). Moreover, similar phenomena were observed in stereomicroscopic images of cartilage‐hydrogel constructs after overnight incubation at 37 °C (Figure S3, Supporting Information). Furthermore, the increase of polymer solid content (GelMA 16%, w v^−1^) did not yield a robust integration between the hydrogel and cartilage in either of the photoinitiator groups, underscoring the combined influence of GC and Ru/SPS cross‐linking mechanisms (Figure S3C,F, Supporting Information). The joint is expected to be protected from loading immediately after surgery; however, the ex vivo joint study demonstrated that Ru/SPS cross‐linked GelMA‐GC hydrogel could withstand minor loading conditions and maintain secure integration once applied in vivo. These results suggest that the Ru/SPS cross‐linked GelMA‐GC hydrogel may be a viable option for protecting defects from loading while allowing them to remain functional.

In this study, we focused on evaluating the short‐term durability and adhesive properties of hydrogels in a joint context. Although the findings showed promise for use, further in vitro and in vivo studies are required to address the critical issue of long‐term joint lubrication. Future in vivo studies can provide valuable insights into the hydrogel's behavior in a more realistic setting, considering the dynamic nature of joint movement and the presence of synovial fluid.

### 2.4 GelMA‐GC Hydrogel Precursor Solution Is Injectable

**Figure 4:**
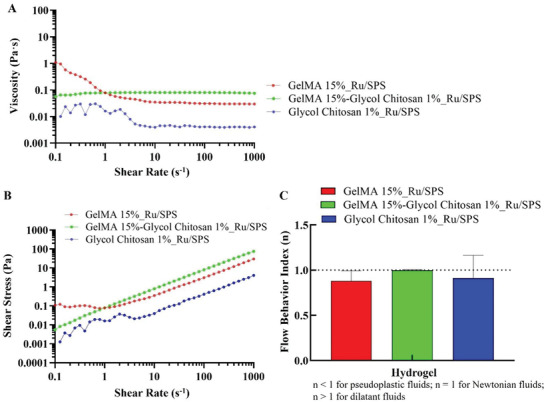
Rheological characterization of GelMA and GelMA‐GC hydrogel precursor solutions: flow curve. Flow curve of GelMA (15%, w v^−1^), GelMA‐GC (15% GelMA and 1% GC, w v^−1^), and GC (1%, w v^−1^) polymer solutions with Ru/SPS photoinitiators. A) shear dependent viscosity, B) shear stress versus shear rate, and C) flow index (*n*) measured at 37 °C. Sample size *n* = 3 per group; error bars: Mean ± SD.

Rheological properties of GelMA (15%, w v^−1^), GelMA‐GC (GelMA:GC; 15:1%, w v^−1^), and GC (1%, w v^−1^) solutions containing Ru/SPS at 37 °C were monitored to determine the effect of different shear rates ranging from 0.1 to 1000 s^−1^ on shear stress and viscosity. The viscosity of GelMA and GC hydrogel precursor solutions reduced in the low shear rate range (≈0.1–30 s^−1^) indicating molecular rearrangement of polymer in the flow direction. Both hydrogel precursor solutions showed a Newtonian plateau at high shear rate afterwards (**Figure** **4**A). GelMA‐GC hydrogel precursor solution, on the other hand, exhibited Newtonian behavior over the whole flow curve despite having a higher viscosity than GelMA and GC hydrogel precursor solutions (Figure 4A). The Newtonian behavior of GelMA, GelMA‐GC, and GC hydrogel precursor solutions was also observed in the shear stress versus shear rate curve (Figure 4B), where the shear stress of all hydrogel precursor solutions increased continuously as the shear rate increased. In addition, we characterized the behavior of the hydrogel precursor solution by fitting the Power Law equation [Equation (3)] to the linear component of each material's shear rate–viscosity rheology plot, where they also exhibited Newtonian behavior (Figure 4C).

Injectable hydrogels containing cells and bioactive materials can be used to properly fill irregularly shaped defects using minimally invasive techniques.^[^ ^70^ ^]^ We studied the injectability of the GelMA_Ru/SPS and GelMA‐GC_Ru/SPS hydrogel precursor solutions based on viscosity, given the encouraging results of the mechanical and adhesion tests. Viscosity is a fluid's resistance to flow or the amount of friction that exists in the material. The greater the viscosity, the more friction exists, so more pressure is required to inject it. As injection will be performed manually, there will be a chance of a variable injection rate. In this case, the injection of hydrogel precursor solution will be more manageable if the viscosity remains constant throughout the injection rate, as described by a fluid's Newtonian behavior in rheology. Although the viscosity of the GelMA solution increased with the addition of GC, the flow curve analysis revealed its Newtonian behavior (Figure 4A). We also observed the Newtonian behavior of the GelMA‐GC_Ru/SPS hydrogel precursor solution on the flow index values of the power law equation (Figure 4C). Yoshida et al.^[^ ^71^ ^]^ observed the strongest correlations between viscosity at a shear rate of 10^3^ s^−1^ and injection pressure of submucosal injection materials. Our hydrogel precursor solutions exhibited Newtonian behavior at that shear rate and was effectively delivered in the tensile test model using a 1 mL syringe with a 19‐gauge needle at 37 °C, further verifying the injectability of this system. However, due to Newtonian behavior, injecting cell‐laden GelMA‐GC hydrogel precursor solution at a higher rate poses a risk of increased shear stress on encapsulated cells, which could lead to cell death and needs future attention. Moreover, the rheological analysis demonstrated the unsuitability of the GelMA‐GC precursor solution for bioprinting, where shear‐thinning behavior is critical for a bioink to be extruded through a small orifice.^[^ ^72^ ^]^

### 2.5 GelMA‐GC Hydrogels Cross‐Linked by Ru/SPS Are Degradable

**Figure 5:**
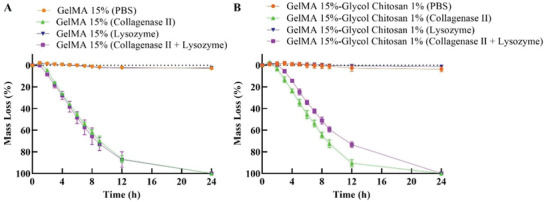
Enzymatic degradation of Ru/SPS cross‐linked A) GelMA (15%, w v^−1^) and B) GelMA‐GC (15% GelMA and 1% GC, w v^−1^) hydrogels based on the mass loss of hydrogels at different time points for 24 h as static culture at 37 °C. Sample size *n* = 6 per group; error bars: Mean ± SD.

A degradation test of Ru/SPS cross‐linked GelMA and GelMA‐GC hydrogels was carried out using collagenase II and lysozyme enzymes as a static culture at 37 °C after getting the swelling equilibrium. Hydrogels kept in collagenase solution significantly reduced weight compared to control (**Figure** **5**). All GelMA and GelMA‐GC hydrogels kept in collagenase or collagenase + lysozyme solutions lost weight gradually over the first 12 h and were entirely dissolved after 24 h. However, GelMA and GelMA‐GC hydrogels maintained in both PBS and lysozyme solutions did not degrade and remained unchanged for at least 24 h (Figure 5A,B).

Biodegradability has gained considerable attention in tissue engineering applications as a desirable feature of hydrogel materials as the degraded hydrogels will allow the cells to reorganize and restructure their surroundings with their own ECM. Hydrogels synthesized from natural substrates such as collagen, chitosan, gelatin, fibrin, and hyaluronan are shown to be biodegradable.^[^ ^73^ ^]^ Matrix metalloproteinases (MMPs) are a class of zinc‐dependent endopeptidases that govern the breakdown of ECM proteins.^[^ ^74^ ^]^ MMPs are typically either released from cells or bound to the plasma membrane by proteoglycans such as heparan sulfate glycosaminoglycans.^[^ ^75^ ^]^ Since MMPs play a crucial role in ECM remodeling, they are abundant in most connective tissues. Collagenases are members of the MMP family that can degrade and remodel the extracellular matrix for cell spreading and migration.^[^ ^76^ ^]^ Collagenases include MMP‐1 (interstitial collagenase), MMP‐8 (neutrophil collagenase), MMP‐13 and MMP‐18.^[^ ^74^ ^]^ In particular, GelMA retains target sequences of MMPs^[^ ^77^ ^]^ such as collagenases and upon implantation undergoes proteolytic degradation. ^[^ ^78^ ^]^ Therefore, cells encapsulated in GelMA hydrogels can degrade and remodel the surrounding hydrogel by replacing it with cell‐secreted ECM.^[^ ^77^, ^79^ ^]^ On the other hand, lysozyme is the only enzyme found in human serum able to degrade chitosan by enzymatic hydrolysis of the glycosidic bonds of acetylated residues on the GC backbone.^[^ ^80^ ^]^ Neutrophils and macrophages can also release lysozyme in response to acute/chronic inflammation arising from an injury or surgical procedure.^[^ ^81^ ^]^ Herein, collagenase and lysozyme both were used to examine the degradability of Ru/SPS cross‐linked GelMA and GelMA‐GC hydrogels (Figure 5). All GelMA containing hydrogels (with or without GC) showed complete degradation by collagenase which may be due to the presence of MMP‐sensitive motifs in the GelMA backbone. ^[^ ^82^ ^]^ As expected, GelMA and GelMA‐GC hydrogels incubated in control PBS exhibited no or minimal degradation (≈4%–5%) after 12 h (Figure 5). A similar trend was also observed in GelMA hydrogels kept in lysozyme solution (Figure 5A). Therefore, it may be concluded that GelMA hydrogels are not biodegradable under the hydrolytic action of lysozyme due to the absence of glycosidic linkages and that the hydrogel weight loss may be a result of matrix degradation by PBS. Tylingo et al. also described a similar behavior of collagen and gelatin during the production of the chitosan‐collagen‐gelatin composite scaffold.^[^ ^83^ ^]^ Contrary to our expectations, even with the lysozyme solution, GelMA‐GC hydrogels degraded similarly to when exposed to PBS, showing minimal or no lysozyme‐mediated degradation (Figure 5B). This may be due to the low concentration of GC present in the hydrogels, where the breakage of glycosidic linkages in the GC backbone may not have resulted in a significant weight loss. While the in vitro degradation test employed a collagenase concentration exceeding physiological levels, the degradation of the hydrogels indicates the biodegradability of both hydrogel systems. This suggests their potential to be replenished by extracellular matrix (ECM) released by encapsulated cells within an in vivo condition.

### 2.6 GelMA‐GC Hydrogels Cross‐Linked by Ru/SPS Are Cytocompatible and Biochemically Conducive to Chondrogenesis

**Figure 6:**
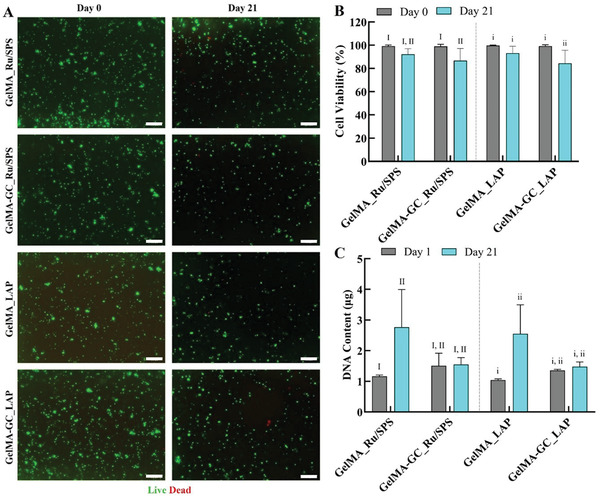
Viability of bovine articular chondrocytes in GelMA (15%, w v^−1^) and GelMA‐GC (15% GelMA and 1% GC, w v^−1^) hydrogels cross‐linked by Ru/SPS and LAP photoinitiators. A) Representative live/dead images of chondrocytes encapsulated (seeding density: 1.6 × 10^6^ cells mL^−1^ of hydrogel) in GelMA and GelMA‐GC hydrogels. Living cells appear green, dead cells appear red. B) Cell viability represented as a percentage on day 1 and day 21 of culture. C) Total DNA content of hydrogel constructs on day 21. Scale bars: 200 µm. Sample size for live/dead *n* = 3 and DNA quantification *n* = 5 for each group, error bars: Mean ± SD. Groups that do not share a common Roman numeral are statistically different (*p* < 0.05). The comparison was conducted just inside each cross‐linker group, as indicated by capital and lowercase numerals. GelMA (15%, w v^−1^) hydrogels were considered as control.

To determine the cytocompatibility of GelMA and GelMA‐GC hydrogels, we conducted a 21‐d cell viability study. We encapsulated bovine passage one (P_1_) chondrocyte into GelMA and GelMA‐GC hydrogels and cross‐linked them with Ru/SPS and LAP photoinitiators for 5 and 3 min, respectively, at 405 nm. Hydrogels were cultured in chondrogenic differentiation media for 21 d, and cell viability was evaluated using the live/dead assay on day 0 and day 21 as described earlier.^[^ ^84^ ^]^ The cell viability was high (>85%) but reduced slightly in all groups on day 21 of cell culture (**Figure** **6**A,B).

**Figure 7:**
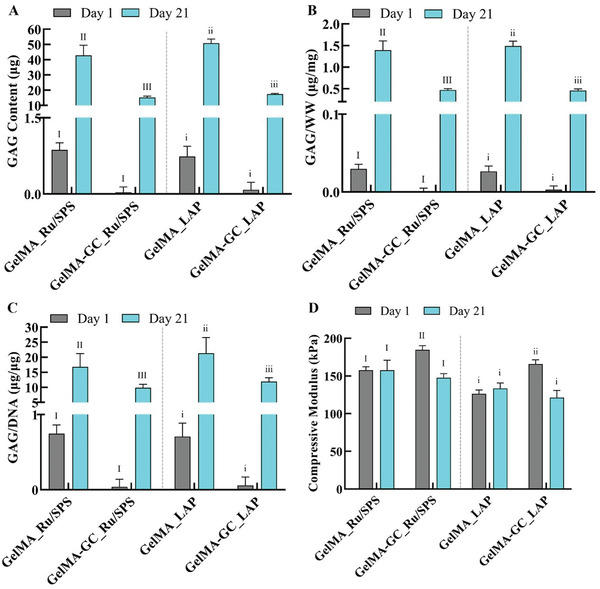
Biochemical and mechanical properties of GelMA (15%, w v^−1^) and GelMA‐GC (15% GelMA and 1% GC, w v^−1^) hydrogels cross‐linked by Ru/SPS and LAP photoinitiators at 405 nm at day 21 of cell culture. Bovine articular chondrocytes were encapsulated (seeding density: 1.6 × 10^6^ cells mL^−1^ of hydrogel) in GelMA and GelMA‐GC hydrogels and cultured at 37 °C with 5% CO_2_ for 21 d with media change twice in a week. A) Total GAG content and B) total GAG content normalized to wet weight. C) Total GAG content normalized to total DNA content and D) compressive modulus (CM); *n* = 5 per group, error bars: Mean ± SD. Groups that do not share a common Roman numeral are statistically different (*p* < 0.05). The comparison was conducted just inside each cross‐linker group, as indicated by capital and lowercase numerals. GelMA (15%, w v^−1^) hydrogels were considered as control.

We determined the GAG and DNA content on days 1 and 21 by DMMB and PicoGreen assays, respectively. In Ru/SPS cross‐linked groups, GelMA‐GC hydrogels showed comparable DNA content to GelMA hydrogels on day 21. Similar phenomena were observed in LAP groups on day 21 of culture (Figure 6C). When DNA content was normalized to the wet weight (WW) of hydrogels, GelMA‐GC hydrogels maintained similar levels over the three weeks of culture; however, GelMA hydrogel DNA/WW approximately doubled (Figure S4D, Supporting Information). The GAG content in all GC‐containing hydrogels cross‐linked by both photoinitiators was lower than that of the GelMA hydrogels on day 21 of culture (**Figure** **7**A). Similar phenomena were also observed when GAG content was normalized to the wet weight of hydrogels (Figure 7B). On day 21, a significant increase of GAG/DNA content was observed in both Ru/SPS, and LAP cross‐linked GelMA‐GC hydrogels compared to their day 1 controls; however, it was significantly lower compared to day 21 GelMA controls in their respective groups (Figure 7C).

We also determined the mechanical properties of chondrocyte‐laden GelMA and GelMA‐GC hydrogels by conducting unconfined uniaxial compression tests at day 1 and 21 of cell culture by following the same protocol as described in the previous section. On day 21 of culture, the Ru/SPS cross‐linked GelMA‐GC hydrogels showed a comparable CM (147.5 ± 5.6 kPa) to the control (156.0 ± 15.2 kPa). Similar trends were observed in LAP cross‐linked hydrogels, where GelMA‐GC and GelMA hydrogels exhibited CM values of (121.3 ± 9.5 kPa) and (133.4 ± 7.3 kPa), respectively (Figure 7D). CM was maintained in GelMA hydrogels cross‐linked with Ru/SPS and LAP over 21 d of culture. However, CM decreased in GC hydrogel groups cross‐linked with Ru/SPS and LAP cross‐linkers over the same time‐period, to similar levels as the GelMA hydrogels (Figure 7D).

The viability of the encapsulated bovine articular chondrocytes (bAC) was determined based on the total live/dead cell counts in the sample (Figure 6A,B). The biocompatibility of GelMA and GC was previously explored, and both are suitable for chondrocyte growth and proliferation.^[^ ^85^ ^]^ The cell‐laden GelMA and GelMA‐GC hydrogels cross‐linked by LAP and Ru/SPS photoinitiators in this study revealed good vitality and abundant living cells after 21 d of in vitro culture (Figure 6A,B). Chondrocytes typically have a rounded morphology in a 3D environment, which is indicative of their chondrogenic nature.^[^ ^86^ ^]^ At each time point, it was noted that the encapsulated cells in our hydrogel system were uniformly dispersed and retained their round shape (Figure 6A). Pahoff et al. found that human chondrocytes had high cell viability in GelMA cross‐linked with LAP, which was maintained over 28 d.^[^ ^84^ ^]^ In a separate investigation, Lim et al. showed high cell viability (>80%) of human articular chondrocytes in LAP and Ru/SPS systems.^[^ ^41^ ^]^ Despite using a higher concentration of LAP and Ru/SPS than Lim et al.,^[^ ^41^ ^]^ the cell viability of bovine chondrocytes was >85% at day 21 culture in both photoinitiator groups (Figure 6B). We used the same concentration and cross‐linking conditions for the LAP group as Pahoff et al.^[^ ^84^ ^]^ study and observed high cell viability in the LAP cross‐linked hydrogels. All these observations imply that our hydrogel system cross‐linked by both Ru/SPS and LAP offers a cytocompatible environment.

To visualize the internal structures and pore sizes of the hydrogels, we performed scanning electron microscopy (SEM) following a preestablished protocol.^[^ ^87^ ^]^ However, the processing of hydrogel samples from both photoinitiator groups for SEM imaging resulted in substantial sample shrinkage, causing disruption to the inherent architecture of the hydrogel. Moreover, due to the limited sample availability and the extensive structural deterioration of the hydrogel structure due to the removal of water content during the freeze‐drying process, images obtained via SEM analysis were deemed inadequate for true quantification of hydrogel pore size due to significant shrinkage. Therefore, future investigation is necessary to develop a viable approach for SEM imaging customized to this type of hydrogel. Nevertheless, from the SEM images acquired, qualitative observations exhibited comparable pore characteristics within both hydrogel systems (Figure S5, Supporting Information). Although direct analysis of pore size was not performed, the cell viability and proliferation data presented herein strongly suggest that the pore structure of this hydrogel system is conducive to cellular growth and proliferation.

To determine the cell proliferative potency, we measured the DNA content at days 1 and 21 by PicoGreen assay (Figure 6C). In the Ru/SPS group, GelMA‐GC hydrogels had a DNA content comparable to that of GelMA at day 21 of culture. A similar trend was observed in the LAP cross‐linked GelMA‐GC hydrogels compared to GelMA hydrogels (Figure 6C), indicating a slow rate of cellular proliferation. Complexation between the positively charged GC and negatively charged DNA can be formed, a phenomenon that is affected by both concentration and pH. ^[^ ^88^ ^]^ Therefore, we determined DNA content of GC containing hydrogels utilizing a multiplying factor, however, we assume that this method did not completely solve the issue of GC‐mediated interactions with the DNA standard. Therefore, further research is needed to understand the interactions between GC and DNA standards and to develop strategies to circumvent this effect. Although all GC‐containing hydrogels produced significantly less GAG at day 21 compared to their respective GelMA controls at day 21, GAG accumulation was significantly higher compared to their day 1 controls in both groups (Figure 7A). Furthermore, the synthesis of GAGs in our hydrogel system implies that it is suitable for tissue growth. Lim et al.^[^ ^41^ ^]^ cross‐linked 10% (w v^−1^) GelMA with 0.2/2 (mM/mM) Ru/SPS or 0.05 wt% LAP for 15 min at 30 mW cm^−2^ (400–450 nm) and found significantly greater GAG content in both samples compared to those cross‐linked with the UV + I2959 system (0.05 wt% I2959). Pahoff et al.^[^ ^84^ ^]^ similarly found a significant increase in GAG accumulation in LAP cross‐linked hydrogels on day 28 compared to day 1 with 10% (w v^−1^) GelMA cross‐linked for 2.5 min at light intensity of 10 mW cm^−2^ at 405 nm. We employed 15% (w v^−1^) GelMA with 0.5/20 mm/mm Ru/SPS or 0.15% (w v^−1^) LAP and cross‐linked the hydrogels for 5 or 3 min, respectively, at light intensity of 9 mW cm^−2^ at 405 nm with a cell population of 1.6 × 10^6^ cells mL^−1^ of hydrogel. Considering the re‐differentiation capacity of cell‐encapsulated hydrogels, the ratio of GAG/DNA in Ru/SPS and LAP cross‐linked GelMA‐GC samples on day 21 was significantly higher than that of their respective GelMA samples on day 1 (Figure 7C). At day 21 however, the GAG synthesis of GelMA‐GC hydrogels in both groups was significantly decreased compared to their respective GelMA controls of day 21 (Figure 7C). GAG bind and retain water inside the ECM of articular cartilage, aiding its resistance to fluid‐driven compressive strain.^[^ ^89^ ^]^ Hydrogel stiffness appears to be correlated to the accumulation of GAG, unlike other ECM components. We hypothesized that the lower GAG content in GelMA‐GC hydrogels compared to GelMA hydrogels on day 21 would significantly reduce the CM compared to their respective controls. Contrary to our expectations, the reduction of GAG did not substantially affect the CM of GelMA‐GC hydrogels at day 21 relative to their respective control (Figure 7D), suggesting that both the GAG accumulation and molecular interactions are likely contributing to the compressive stiffness of the GelMA‐GC hydrogels. The lower GAG accumulation in GelMA‐GC hydrogels cross‐linked by both photoinitiators may be attributable to the lower number of viable cells (Figure S4E, Supporting Information) present in the hydrogels on day 21; however, additional research is required to confirm this hypothesis.

Highly hydrophilic GAGs are readily released from tissue‐engineered constructs.^[^ ^90^ ^]^ We observed that a significant quantity of GAG was released in the media of LAP cross‐linked GelMA and GelMA‐GC hydrogels. High levels of GAG were also detected in medium containing Ru/SPS‐cross‐linked GelMA hydrogels (Figure S4A,B, Supporting Information). This may be related to the hydrogel stiffness in those groups that failed to retain accumulated GAG within the hydrogels. However, Ru/SPS cross‐linked GelMA‐GC hydrogels and their medium contained lower quantities of GAG than the control (Figure S4A,B, Supporting Information). This may be summed up in two aspects: one may be due to the hydrogel stiffness preventing GAG release from the constructs to the media. Another may be due to the ionic interactions between GC and Ru^3+^ generated during Ru/SPS cross‐linking or the anionic cell membrane, which further interfered with the GAG production from cells. However, further study is required to resolve this assumption.

The most essential requirements for an adhesive to be suitable for cartilage repair are adequate mechanical and adhesive properties, an appropriate swelling and degradation profile, and biocompatibility. Bioadhesives for cartilage repair must be mechanically robust to promote cartilage regeneration while preserving the phenotypic and structural characteristics of the newly formed tissue. In addition, the adhesive should be able to integrate with the underlying tissue just after application to initiate the healing process. In this study, Ru/SPS cross‐linked GelMA‐GC hydrogels demonstrated promising mechanical and adhesive properties suitable for cartilage defect repair. Moreover, the *ex vivo* joint study has demonstrated efficacy of this hydrogel system to remain securely attached to the defect even when the joint is subjected to a minor load‐bearing condition. We have also recently demonstrated that this hydrogel remains integrated with surrounding cartilage through two weeks of dynamic compression culture, and that embedded chondrocytes express chondrogenic markers COL2A1 and ACAN.^[^ ^91^ ^]^ From these observations, we anticipate that this biodegradable and injectable hydrogel system will offer secure integration with native cartilage after in vivo application. The encouraging cell survival, GAG, and DNA synthesis of this bioadhesive hydrogel points to its potential as an alternative to existing bioadhesive formulations. However, further in vitro and in vivo studies are warranted to fulfill this hypothesis.

Our study is not devoid of limitations. There is a limited supply of chondrocytes from healthy human donors; hence patients undergoing total knee arthroplasties for OA are typically the source of these cells. Consequently, cells are likely affected by pathology, in contrast to individuals without degenerative joint disease. We utilized fresh animal‐derived cell sources in response to this circumstance. In addition, to evaluate the adhesive characteristics of our hydrogel system, we required a substantial amount of fresh articular cartilage, which is not easily attainable with human tissues. As bovine articular cartilage is a recognized model for human cartilage of comparable thickness,^[^ ^92^ ^]^ bovine chondrocytes and articular cartilage were utilized throughout our investigation. However, getting appropriate and fresh samples following animal slaughter from farms or butcher shops proved difficult and time‐consuming, a factor that should be considered in future research. Although the tensile test methodology enables evaluation of the adhesive strength of hydrogels while adhering to cartilage construct, we cannot completely rule out the possibilities of hydrogel dislodgement from the construct during silicone tube removal. Therefore, it is crucial to ensure sufficient caution when removing the silicone tube prior to the tensile test. While satisfactory hydrogel adhesion was claimed in the joint movement study, the results were only presented qualitatively. Future studies will concentrate on the quantitative analysis of the adhesive strength of GelMA‐GC bioadhesive hydrogels in a joint context.

## 3 Conclusion

Bioadhesive hydrogels can circumvent the limitations of current cartilage tissue engineering approaches. This study describes the synthesis of a GelMA‐based injectable bioadhesive hydrogel with promising mechanical properties using visible light photo‐polymerization and glycol chitosan incorporation. The results indicate that Ru/SPS cross‐linked GelMA‐GC hydrogels are biocompatible, biodegradable, highly adhesive, and have the potential as an injectable hydrogel for cartilage tissue engineering. Additional in vitro and in vivo studies are needed to demonstrate the efficacy of this hydrogel system.

## 4 Experimental Section

### Hydrogel Preparation

Sterile freeze‐dried GelMA polymer (gelatin methacryloyl, porcine skin, type A, 80% degree of functionalization, Gelomics Pty Ltd., Australia) was dissolved in autoclaved phosphate buffered saline (PBS) (Thermofisher Scientific, Australia) at 35% (w v^−1^) for 24 h at 37 °C in a thermoshaker with continual shaking. GC (≥60% titration, crystalline, Sigma‐Aldrich, USA) was dissolved in PBS to obtain a 3% (w v^−1^) stock solution and then filter sterilized. Stock solutions of all photoinitiators (50 × 10^−3^ m Ru (tris (2,2′‐bipyridyl) dichlororuthenium (II) hexahydrate ([RuII(bpy)_3_]^2+^), 1 M SPS (sodium persulfate), and 3% (w v^−1^) LAP (lithium acylphosphinate) (all Sigma‐Aldrich, USA) were prepared in PBS and sterile‐filtered (Filtropur S 0.22, Sarstedt, Germany) on the day of hydrogel construct preparation. Hydrogel precursor solutions of GelMA (15% w v^−1^) and 15% w v^−1^ GelMA with 1% w v^−1^ GC (GelMA‐GC) were prepared in PBS and with either 0.5 × 10^−3^ m Ru/20 × 10^−3^ m SPS or 0.15% w v^−1^ LAP at 37 °C.

The hydrogel construct was fabricated according to the method reported by Loessner et al.^[^ ^93^ ^]^ Briefly, after preparation, hydrogel precursor solutions were transferred into a polytetrafluoroethylene (PTFE) casting mold, covered with a glass slide (both sterilized with 70% (v/v) ethanol) and cross‐linked in a LED crosslinker (Luna Crosslinker, Gelomics, Australia) for 5 min (Ru/SPS) or 3 min (LAP) at 405 nm with an intensity of 9 mW cm^−2^. Hydrogel constructs were cut to sizes (4 mm × 4 mm × 2 mm; length × width × height) with a sterile scalpel and acrylic hydrogel cutting guides. In the tensile and push‐out tests, a 1 mL syringe with a 19‐gauge needle was utilized to inject the hydrogel precursor solution into the application site. GelMA hydrogels were considered as the control in each group.

### Articular Cartilage Ring Preparation

**Figure 8:**
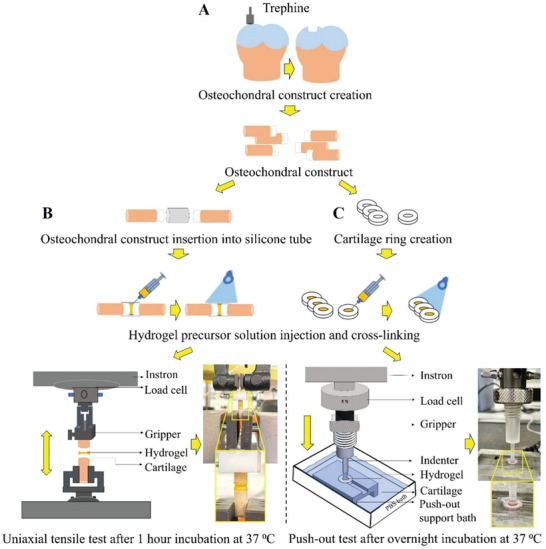
Osteochondral construct preparation and adhesion tests. Schematic representation of adhesion test. A) Osteochondral constructs were harvested using a trephine and used for either B) uniaxial tensile tests (inset illustrates hydrogel failure in between osteochondral constructs during test) or C) push‐out test (inset illustrates the contact position of indenter and hydrogel in PBS bath) on Instron 5567 with 5 N load cell.

Articular cartilage rings were prepared from fresh bovine stifle joints (1–3 years of age) purchased from a butcher shop. The joint was kept moist during transport and extraction by rinsing it with PBS containing penicillin (100 U mL^−1^) and streptomycin (100 µg mL^−1^) (all from Gibco, Thermofisher Scientific, Australia). The femoral condyles and the trochlear groove were trephined to create osteochondral constructs (height: 10–15 mm, diameter: 9 mm). The osteochondral constructs were transferred in a custom‐made mold to remove the superficial cartilage layer with a sterile scalpel. After that, a custom‐made mold and sterile scalpel were used to slice cartilage discs (thickness ≈ 1.6 mm) from the osteochondral constructs (**Figure** **8**; Figure S6A, Supporting Information). All cartilage discs were prepared in an aseptic manner and stored at −20 °C. Prior to use, the discs were thawed for 10 minutes at 37 °C in PBS containing penicillin (100 U mL^−1^) and streptomycin (100 µg mL^−1^). A 4‐mm biopsy punch (Kai Medical, Japan) guided by a custom‐made mold was used to produce a central defect in each cartilage slice (Figure S6B,D, Supporting Information). The cartilage rings (thickness ≈ 1.6 mm, outer diameter: 9 mm, inner diameter: 4 mm) were then kept sterile in PBS containing penicillin (100 U mL^−1^) and streptomycin (100 µg mL^−1^) at room temperature until use.

### Cell‐Free Cartilage–Hydrogel Construct Preparation

The hydrogel precursor solutions of GelMA and GelMA‐GC were prepared as outlined in the section of hydrogel preparation. The cell‐free hydrogel precursor solution was injected into the central defect (diameter: 4 mm) of cartilage rings prepared in the “articular cartilage ring preparation” section. Hydrogel precursor solution leakage or overflow was prevented by placing the cartilage rings in a polydimethylsiloxane (PDMS) sheet with barriers and cross‐linking in an LED cross‐linker (Luna Crosslinker, Gelomics, Australia) for 5 min (Ru/SPS) or 3 min (LAP) at 405 nm. The samples were transferred in the 48‐well plates (Thermofisher Scientific, Australia) containing PBS (pH = 7.4) and incubated at 37 °C for 24 h.

### Mechanical Properties

*Compression Testing*: The CM of the hydrogels was determined using an optimized unconfined uniaxial compression test protocol.^[^ ^93^ ^]^ Briefly, freshly prepared hydrogels (*n* = 6) were weighed before and after 24 h incubation at 37 °C in PBS (pH = 7.4). The cross‐sectional area of hydrogels was calculated using Image J software (National Instruments, USA) from stereomicroscopic (Nikon SMZ745T, Japan) images of hydrogels taken before the compression test. A compression test was conducted using an Instron 5567 (Instron 5567, USA) with a nonporous indenter fitted in a 500 Newton (N) load cell in a custom‐made immersion bath filled with PBS (pH = 7.4) at 37 °C.^[^ ^93^ ^]^ The strain rate was set at 0.01 mm s^−1^ until >15% compressive strain was reached. The height of each hydrogel was determined from the force–displacement curve as the point at which force started to deviate significantly from zero.^[^ ^93^ ^]^ The CM was calculated as the slope of the linear region between 10% and 15% strain on the stress–strain curve.

*Swelling Ratio*: The equilibrium swelling of hydrogels was determined, as described elsewhere.^[^ ^94^ ^]^ Briefly, weights of freshly prepared hydrogels were taken before (*W*_before_) and after 24 h incubation (*W*_after_) with PBS at 37 °C (pH = 7.4) and the swelling ratio was calculated as follows

(1) $$\mathrm{Swelling}\;ratio\left(\mathrm{SR}\right)=\left\{\left(W_{\mathrm{after}}-W_{\mathrm{before}}\right)/W_{\mathrm{before}}\right\}\times 100\%$$

*Microindentation Test*: The mechanical characteristics of cell‐free hydrogel constructs were evaluated by microindentation compression testing after overnight incubation at 37 °C in PBS as described elsewhere. ^[^ ^95^ ^]^ Using a high‐precision piezoelectric actuator‐controlled microcompression system (CellScale Biomaterials Testing, Canada), microindentation at a constant jogging speed (4 µm^−1^ s) was conducted at 37 °C in a water bath containing PBS (pH = 7.4). Briefly, a cartilage–hydrogel construct was placed on a testing anvil and the microbeam was positioned just above the center of the sample (Figure 1C). The hydrogels were indented with a 0.5 mm Zirconium oxide bead (ZROB05, Next Advance, USA), which was attached to the ends of 0.5588 mm diameter cantilevered steel microbeams (Figure 1C). Cyclic compression cycles were generated by the microbeam attached to the beads consisting of a vertical force with amplitudes of 10% of the sample height. Each cycle commenced with a load phase (30 seconds) and ended with a recovery phase (30 s) without resting time. Three cycles were performed for each hydrogel sample (*n* = 4). During the experiment, the indentation force (*F*) and indentation depth (*δ*) were continually estimated based on the optically recorded deflection of the cantilevered beam's indenter end and the piezo‐controlled *z*‐displacement of the cantilevered beam's fixed end. Upon compression, the change in hydrogel thickness was monitored, and the jogging force needed to generate a force–displacement curve was recorded. Using the Hertz contact model^[^ ^37^ ^]^ for a spherical indenter, experimental force–indentation depth data were fitted to determine the sample's elastic modulus (*E*) by the following equation

(2) $$E=\frac{3\left(1-v^{2}\right)F}{4R^{0\cdot 5}\delta^{1\cdot 5}}$$

where *R* is the radius of the indenter and ν is the Poisson's ratio (assumed to be 0.44).^[^ ^96^ ^]^ Samples were utilized for push‐out test immediately after mechanical loading.

### Adhesive Properties

*Uniaxial Tensile Test: Osteochondral Construct Preparation*: The adhesive strength of hydrogels was determined by uniaxial tensile test utilizing bovine osteochondral constructs. Osteochondral constructs (height: 15–20 mm, diameter: 5 mm) were harvested from the medial and lateral femoral condyles by a trephine (Figure 8A). The superficial layer of cartilage was removed, and two osteochondral constructs were inserted in a medical‐grade silicone tube (length: 10 mm, inner diameter: 5 mm, wall thickness: 0.6 mm; Gecko Optical, Australia) with the cartilage facing each other, leaving about 1 mm gap between them (Figure 8B). The freshly prepared hydrogel precursor solution was injected into the gap by a 1 mL syringe equipped with a 19‐gauge needle and cross‐linked either in a LED crosslinker (Luna Crosslinker, Gelomics, Australia) for 5 min (Ru/SPS) or 3 min (LAP) at 405 nm. After cross‐linking, the constructs were incubated at 37 °C with PBS (pH = 7.4) for 1 h.

*Adhesive Strength Determination: Tensile Test*: A uniaxial tensile test was performed to determine hydrogel adhesive strength with a 100 N load cell in an Instron 5567 after 1 h incubation at 37 °C. The upper end of the osteochondral constructs was clamped with a custom‐made acrylic grip attached to the upper load cell gripper (Figure 8C). The adhesion test was conducted after removing the silicone tube using a scalpel. The strain rate was set at 0.01 mm s^−1^, and the tensile modulus (TM) was determined at 0–3% strain. Four osteochondral constructs (*n* = 4) were used for each group and condition. To quantitatively determine the adhesive or cohesive failure of hydrogels in the osteochondral constructs after the adhesion test, the length (mm) of adherent hydrogels from the nearest cartilage tissue was measured from stereomicroscopic images by Image J software (National Instruments, USA) and subsequently compared. Three areas of interest in the stereomicroscopic images of the osteochondral constructs were selected for measuring the length (two corners and center). The length of hydrogels in osteochondral constructs without adherent hydrogels was considered as 0 mm.

*Push‐Out Test*: The adhesive strength of Ru/SPS and LAP cross‐linked GelMA and GelMA‐GC hydrogels to the articular cartilage was evaluated by conducting a push‐out^[^ ^35^, ^62^ ^]^ test in cell free free‐swelled cartilage‐hydrogel samples after overnight incubation at 37 °C. The test was performed at 37 °C with an Instron 68TM‐30 (Instron 68TM‐30, USA) electromechanical test device equipped with a 5 N load cell at a speed of 0.01 mm s^−1^. Prior to the test, images of cartilage–hydrogel constructs were taken by stereomicroscopic (Stereoscope_NikonBoomStand, Nikon SMZ745T, Japan). To dislodge the cross‐linked hydrogel from the cartilage ring, a 3D‐printed indenter (printed with Biomed Clear printed on a Form 3B+ printer, FormLabs, USA) with a diameter of 3.5 mm were used in the push‐out test (Figure 8C and Figure S6E, Supporting Information). Samples were held securely in place using a custom 3D‐printed support stage submerged in an immersion bath containing PBS at 37 °C (Figure 8C and Figure S6F, Supporting Information). The rim of the upper plate hole confined the cartilage ring during testing. The contact area between the hydrogel and the inner surface of the cartilage defect was calculated as the cartilage defect's circumference multiplied by the hydrogel's height. The push‐out strength was determined by normalizing the maximum force to remove the hydrogel from the cartilage ring to the contact area. Four cartilage rings (*n* = 4) were used for each group.

*Ex Vivo Joint Durability Test*: An ex vivo model was used to test the durability of GelMA and GelMA‐GC hydrogels cross‐linked by Ru/SPS or LAP in a joint environment. Bovine stifle joints (collected from a butcher) were positioned and clamped on the operating table to mimic the joint alignment. The joint capsule was opened for optimal visibility of the femoral condyles, and the surrounding tissues were incised. A biopsy punch was used to create circular chondral defects (*n* = 4; diameter: 10 mm, area: 0.8 cm^2^) in the condyles. The cartilage of the defect site was excised up to the subchondral bone (defect depth ≈ 2 mm) (Figure 3B). The chondral defects were filled with GelMA or GelMA‐GC precursor solution containing Ru/SPS or LAP using a 1 mL syringe equipped with a 23‐gauge needle. A portable light (LIGHTFE Keychain Blacklight, UV 395 nm UV light LED source, China) with a custom‐made stand was placed over the defect site, and a fluorinated ethylene propylene (FEP) fluoroplastic film (DuPont Teflon FEP film, USA) attached to the stand prevented hydrogels from overflowing from the defect site during filling while also ensuring cross‐linking by light penetration (Figure 3A ii). The distal femoral end of the bone was positioned over the proximal end of the tibia after cross‐linking for 5 or 3 min for Ru/SPS or LAP respectively. Immediately after hydrogel cross‐linking, the joint was flexed and extended about fifty times mimicking the joint motion. The test was conducted in a fluid free condition (Videos S1 and S2, Supporting Information). Photos were taken before and after the joint movement.

A comparative study of the adhesiveness of Ru/SPS and LAP cross‐linked 15% (w v^−1^) GelMA with or without 1% (w v^−1^) glycol chitosan hydrogels was performed to observe hydrogel–cartilage integration following joint movement study and India Ink (Parker Quink, France) staining. The defect area (diameter: 10 mm, area: 0.8 cm^2^) was painted with blue India ink after the test to visualize the hydrogel–cartilage integration. India ink adheres to fissured cartilage and improves lesion visibility by contrasting the surrounding normal cartilage,^[^ ^97^ ^]^ which can help visualize the hydrogel–cartilage integration by its presence or absence in the interface macroscopically. Briefly, the cartilage surface was painted for 15 s with a 20% (v v^−1^) solution of blue India ink in PBS. Any excess stain was blotted off with a moist cotton swab. Photographs of the stained hydrogel–cartilage interface were taken after the joint movement test, then analyzed using the Image J software to determine the presence of India Ink at the hydrogel–cartilage interface. Adhesive and cohesive failure of hydrogels was observed during and after the test (Videos S1 and S2, Supporting Information). Hydrogel detachment from the defect site was attempted by pushing the fingertip over the hydrogels 20 times (Video S3, Supporting Information).

### Rheology

*Rheology Sample Preparation*: Samples were prepared as described earlier in the section of hydrogel preparation. Only Ru/SPS cross‐linked hydrogels were analyzed by rheometry, based on the promising results of this cross‐linking system in the mechanical and adhesion tests. The photoinitiators were added with the hydrogel solution and protected from light before transferring the sample (230 µL per‐sample; *n* = 3) to the rheometer plate. Mixing was accomplished by pipetting up and down at 37 °C, and bubbles were removed with a brief period of centrifugation (20 s) (Tomy microOne, Japan).

*Rheological Characterization*: The rheological properties of hydrogel precursor solutions were investigated using a compact rheometer (MCR 302, Anton Paar Germany) equipped with a Peltier plate for temperature regulation. The shear rheology study was conducted using a cone plate (CP25‐1) with a diameter of 25 mm, a one‐degree angle, and a fixed gap of 0.05 mm. The temperature was kept constant at 37 °C for all rheological experiments, and the samples were kept in a thermo‐shaker (Eppendorf AG 22331 Hamburg, Germany) at 37 °C before the measurement. No solvent trap was employed as the samples did not dry during the test (verified prior). After reaching the measuring position and sample trimming, all experiments preceded a 2‐min surveillance period to achieve mechanical and temperature equilibrium. The measurements were carried out under an H‐ETD400 hood with the airflow turned off to prevent drying and maintain a constant sample temperature. For each sample, three measurements were taken, and the mean values were reported. Shear rate sweeps (0.01–1000 s^−1^) were conducted to monitor shear thinning behavior and viscosity.

The hydrogel precursor solution's behavior was characterized by fitting the power law equation to the linear component of each material's shear rate–viscosity rheology plot.

(3) $$\eta =K{\dot{\gamma }}^{n-1}$$

where η = viscosity, $\dot{\gamma }$ = shear rate, *K* and *n* = shear thinning coefficients.

In this equation, the authors were interested in the flow index (*n* value) that reflected the material's behavior (*n* < 1 for pseudoplastic fluids, *n* = 1 for Newtonian fluids, and *n* > 1 for dilatant fluids).

### In Vitro Degradation

To simulate hydrogel degradation under in vivo conditions, an in vitro degradation test was performed using collagenase II and lysozyme enzymes. Ru/SPS cross‐linked GelMA and GelMA‐GC hydrogels (4 mm × 4 mm × 2 mm; length × width × height) were tested for the enzymatic degradation based on optimized previously published protocols.^[^ ^98^ ^]^ The hydrogels (*n* = 6) were placed in 0.5 mL PBS (pH 7.4) and incubated for 24 h at 37 °C to get the swelling equilibrium. The samples were transferred in well plates containing 0.5 mL of PBS (pH 7.4), and PBS containing 28 U mL^−1^ collagenase (Gibco Collagenase, Type II powder, Thermo Fischer Scientific, USA), PBS containing lysozyme 10 000 U mL^−1^ (Lysozyme, from chicken egg white, Sigma‐Aldrich, USA), and PBS containing both collagenase and lysozyme and incubated as a static condition at 37 °C in an incubator. Every 30 min for 9 h and at 12 and 24 h the hydrogels were weighed after removal of excessive liquid carefully by delicate tissue wipe (Kimtech Science, USA). The degree of in vitro degradation was calculated as mass loss according to the following formula

(4) $$\mathrm{Mass}\;Loss=\left\{\left(W_{0}-W_{t}\right)/W_{0}\right\}\times 100\%$$

where *W*_0_ is the initial sample weight (the equilibrium swelling) and *W*_t_ is the weight after time *t*.

### Infrared Analysis

FTIR‐attenuated total reflection (ATR) spectra of GelMA, as well as Ru/SPS and LAP cross‐linked GelMA and GelMA‐GC hydrogels, were obtained using a Nicolet iS50 ABX FTIR spectrometer equipped with a KBr beam‐splitter, a single bounce diamond ATR and a DLaTGS detector with KBr window (ThermoFisher Scientific, USA). A minimum of 64× scans was co‐added with a gain of 4 and optical velocity of ≈0.6 generating a nominal 4 cm^−1^ resolution. The resultant spectrum was ATR and background corrected and matched against a spectral library of ≈16 000 spectra in Thermo Omnic v9.0.

### Hydrogel Ultrastructure

The hydrogel ultrastructure was examined using SEM according to an existing protocol.^[^ ^87^ ^]^ In brief, all hydrogels were fixed with 1% (v v^−1^) paraformaldehyde (PFA) at 4 °C for 24 h. Subsequently, the hydrogels underwent three washes with 1× PBS and an additional rinse with Milli‐Q water to eliminate residual fixatives and salts. The hydrogels were then rapidly freeze‐dried upon immersion in liquid nitrogen. The resultant samples were sectioned and affixed onto 0.5 in. SEM pin stubs (Agar Scientific, Stansted, UK) to enable imaging of the internal hydrogel structure. Imaging was performed at magnifications of 5000× and 10000×, at an acceleration voltage of 15.0 kV, utilizing the Hitachi TM3000 SEM (Hitachi High‐Technologies Corporation) (Figure S5, Supporting Information).

### Bovine Articular Chondrocyte Isolation and Expansion

Fresh bovine cartilage samples (1–3 years of age) were obtained from a butcher shop and transported to the laboratory in PBS supplemented with penicillin (100 U mL^−1^) and streptomycin (100 µg mL^−1^) within 1 h of collection. Chondrocytes were isolated from fresh full‐thickness articular cartilage of the lateral and medial femoral condyles, as reported elsewhere.^[^ ^99^ ^]^ Briefly, cartilage was harvested, minced, and digested in low‐glucose Dulbecco's modified Eagle's medium (DMEM) (Gibco, Thermofisher Scientific, Australia) containing collagenase type II (0.15%, w v^−1^) (Worthington Biochemical Corporation, USA) overnight at 37 °C in an incubator. The chondrocyte‐containing solution was filtered using a 100 µm Cell Strainer (BD Biosciences, USA) and centrifuged at 750 *g* for 5 min. Chondrocytes were washed three times by repeatedly centrifuging at 750 *g* for 5 min and resuspending in sterile PBS. The supernatant was finally removed, and the cells were resuspended in 5 mL of chondrocyte growth medium and counted using a hemocytometer. Chondrocytes were stored in liquid nitrogen vapor until use. For expanding the chondrocytes, DMEM supplemented with 2 × 10^−3^ m GlutaMAX, 10 × 10^−3^ m 4‐(2‐hydroxyethyl)‐1‐piperazineethanesulfonic acid (HEPES), 0.1 × 10^−3^ m MEM non‐essential amino acid solution, 50 µg mL^−1^ penicillin/streptomycin, 0.25 µg mL^−1^ amphotericin B (Fungizone) (all from Thermofisher Scientific, Australia), 0.4 × 10^−3^ m l‐proline, 0.1 × 10^−3^ m l‐ascorbic acid (both Sigma‐Aldrich, Australia), and 10% fetal bovine serum (FBS) (Hyclone, USA) was used.

### Cell‐Laden Hydrogel Construct Preparation

For cell‐laden hydrogels, 1.6 × 10^6^ cells were resuspended in the hydrogel precursor solutions and prepared as outlined above. The hydrogel samples were transferred in the cell culture wells containing chondrogenic differentiation media and incubated at 37 °C for 21 d with media change twice in a week. Differentiation media was composed of serum‐free high‐d‐glucose basal chondrocyte medium (composition same as expansion media) supplemented with insulin–transferrin–selenium (ITS‐G) (100× dilution), bovine serum albumin (1.25 mg mL^−1^), dexamethasone (0.1 × 10^−6^ m) (all from Sigma‐Aldrich, USA), and human recombinant transforming growth factor beta‐3 (TGF‐β_3_; 10 ng mL^−1^) (GroPep, Australia).

### Cell Viability

The viability of chondrocytes was determined on day 0 and day 21 using a live‐dead assay described elsewhere.^[^ ^84^ ^]^ Briefly, cell‐laden hydrogel constructs were incubated for 3 min at room temperature in a PBS solution containing 1 µg mL^−1^ fluorescein diacetate (FDA) and 1 µg mL^−1^ propidium iodide (PI; both from Sigma‐Aldrich, USA) after washing with sterile PBS. The samples were washed with PBS, and four z‐stack images were taken by a Zeiss Axio microscope (Carl Zeiss Axio Imager M2, GmbH, Germany) for each sample (*n* = 3) at each time point and analyzed with Image J software. Cell viability is denoted as a percentage of the total number of cells that were alive and calculated by the following formula

(5) $$\mathrm{Cell}\;{\mathrm{viability}}^{\left[100\right]}=\left\{\mathrm{Viable}\;\mathrm{cells}/\left(\mathrm{Viable}+\mathrm{Dead}\;\mathrm{cells}\right)\right\}\times 100\%$$

### Biochemical Analysis

For the biochemical measurement of glycosaminoglycan (GAG) and DNA content, hydrogel constructs were weighed and digested overnight at 56 °C in phosphate‐buffered EDTA (PBE; pH 7.1) containing 0.5 mg mL^−1^ Proteinase K (Thermofisher Scientific, Australia) in a thermo‐shaker (Eppendorf AG 22331 Hamburg, Germany). The amount of GAG in the digested samples was determined using a dimethylmethylene blue (DMMB) assay.^[^ ^101^ ^]^ The absorbances of hydrogel digest were measured with a CLARIOstar microplate reader (BMG Labtech, Australia) at 525 and 595 nm and compared to a chondroitin sulfate (Sigma‐Aldrich, Australia) standard curve at concentrations ranging from 0 to 100 µg mL^−1^ prepared in PBE. At each media change, GAG secreted to the culture medium were also quantified. The GAG content of media was measured using the dimethyl‐methylene blue (DMMB) (pH 3) assay utilizing chondroitin sulfate standard curve at concentrations ranging from 0 to 100 µg mL^−1^ prepared in media. The DNA content of digested hydrogel samples was measured using the Quant‐iT PicoGreen dsDNA quantification assay (Life Technologies, USA) according to the manufacturer's instructions.

The biosynthetic activity of the cells was assessed by normalizing the GAG quantification to the double‐stranded DNA (dsDNA) content determined by a Quant‐iT PicoGreen reagent (Life Technologies, USA) according to the product protocol. Standard curves were prepared at concentrations ranging from 0 to 1 µg mL^−1^ in 1× Tris‐EDTA (TE) buffer (Sigma‐Aldrich, Australia). GAG content was quantified with a CLARIOstar microplate reader (BMG Labtech, Australia) using excitation and emission wavelengths of 525 and 595 nm, respectively, while dsDNA content was quantified by obtaining the fluorescence intensities at an excitation wavelength of 485/20 nm and an emission wavelength of 528/20 nm.

Even with comparable cell viability to GelMA‐only hydrogels, a decrease in the DNA content in GelMA‐GC hydrogels was detected compared to their respective controls in an experiment suggesting a possible interaction between GC and DNA^[^ ^102^ ^]^ standard or the fluorescent dye. To account for this interaction, the normal DNA standard was calibrated with the GelMA‐GC‐containing DNA standard to calculate the multiplying factor to determine the DNA content of GelMA‐GC hydrogels. First, cell‐free GelMA‐GC hydrogels were weighed and digested overnight at 56 °C in phosphate‐buffered EDTA (PBE; pH 7.1) containing 0.5 mg mL^−1^ Proteinase K in a thermo‐shaker. DNA standards were prepared at concentrations ranging from 0 to 1 µg mL^−1^ in 1× Tris‐EDTA (TE) buffer, and GelMA‐GC‐DNA standards were prepared by adding 5 µL of GelMA‐GC hydrogel digest with 95 µL of each DNA standard. Standard curves were generated using the Quant‐iT PicoGreen dsDNA quantification assay according to the manufacturer's instructions. Finally, the multiplying factor for determining the DNA content of GC‐containing hydrogels was determined through the calibration of a DNA standard with the GelMA‐GC‐containing DNA standard.

### Statistical Analysis

All data were analyzed using GraphPad Prism (version 9; GraphPad, USA). Where appropriate, one‐way or two‐way ANOVA with Tukey's honest significant difference post‐hoc test was employed to compare group means. Differences were considered significant when *p* < 0.05 for all tests and are indicated in figures using symbols.

## Conflict of Interest

The authors declare no conflict of interest.

## Author Contributions

S.P., T.J.K., K.S., P.A.T., and C.M. designed the study. T.J.K., K.S., and P.A.T. supervised the project. S.P. performed all the experiments and analyzed the data. J.W.D. and A.W. assisted in image processing and mold preparation. All authors participated in writing, interpreting the analyzed data, and reviewing the manuscript.

## Supporting information

Supporting Information

Supplemental Video 1

Supplemental Video 2

Supplemental Video 3

## Data Availability

The data that support the findings of this study are available from the corresponding author upon reasonable request.
