# Supplementary material for: Photo‐Cross‐Linkable, Injectable, and Highly Adhesive GelMA‐Glycol Chitosan Hydrogels for Cartilage Repair
Source: Adv Healthc Mater. 2023 Oct 10;12(32):2302078. doi: 10.1002/adhm.202302078 (PMC11468424; doi:10.1002/adhm.202302078)
Supplement: Supplementary file 1 — Supporting Information [file ADHM-12-2302078-s004.pdf]

ADVANCED
HEALTHCARE
MATERIALS

Supporting Information

for *Adv. Healthcare Mater.*, DOI 10.1002/adhm.202302078

Photo-Cross-Linkable, Injectable, and Highly Adhesive GelMA-Glycol Chitosan Hydrogels for Cartilage Repair

Sattwikesh Paul, Karsten Schrobback, Phong Anh Tran, Christoph Meinert, Jordan William Davern, Angus Weekes and Travis Jacob Klein**

Supporting information

Supplementary figures

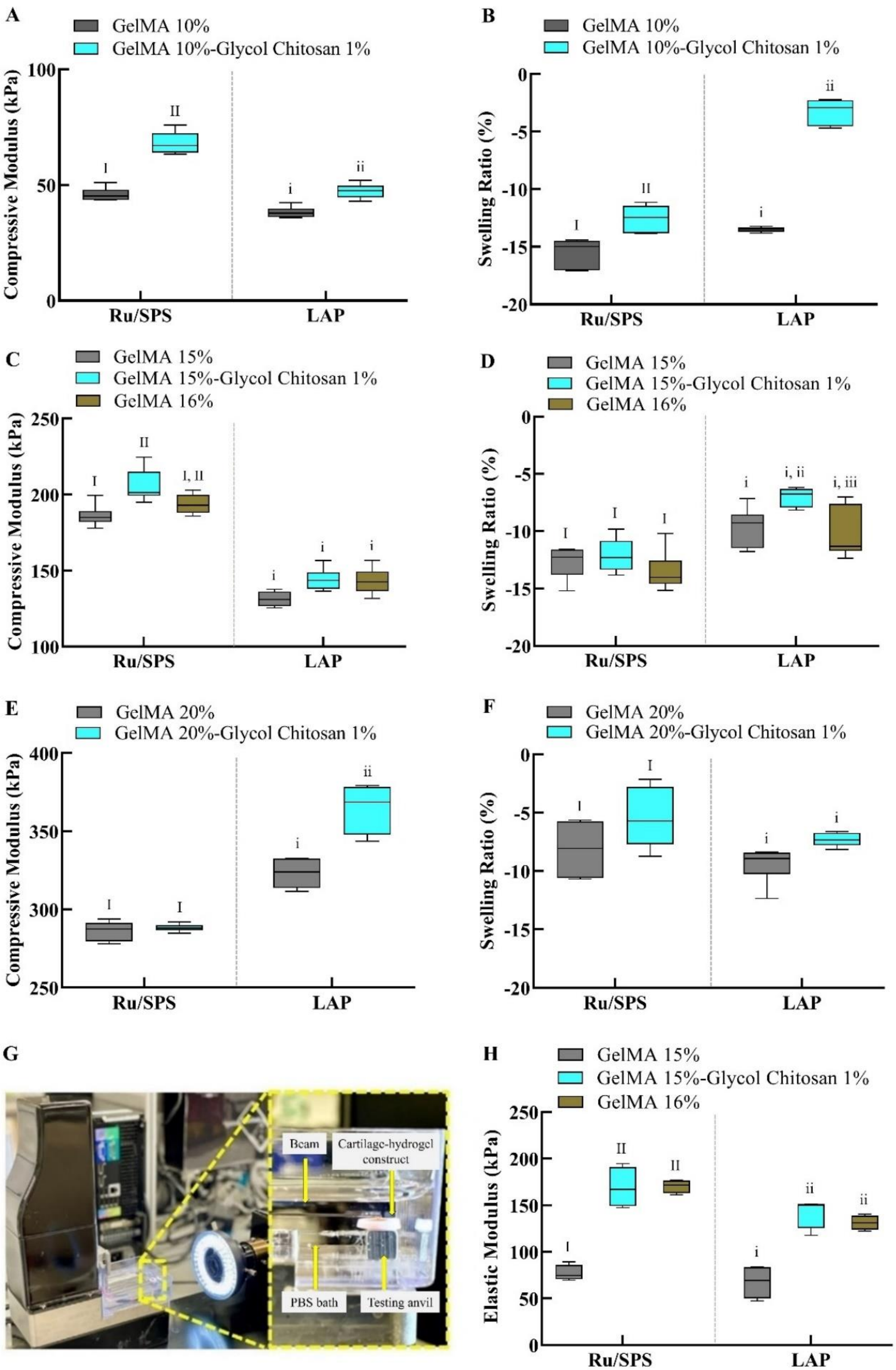
A
GelMA 10%
GelMA 10%-Glycol Chitosan 1%
Compressive Modulus (kPa)
100
50
0
I
II
i
ii
Ru/SPS
LAP
B
GelMA 10%
GelMA 10%-Glycol Chitosan 1%
Swelling Ratio (%)
0
-5
-10
-15
-20
I
II
i
ii
Ru/SPS
LAP
C
GelMA 15%
GelMA 15%-Glycol Chitosan 1%
GelMA 16%
Compressive Modulus (kPa)
250
200
150
100
I
II
I, II
i
i
i
Ru/SPS
LAP
D
GelMA 15%
GelMA 15%-Glycol Chitosan 1%
GelMA 16%
Swelling Ratio (%)
0
-5
-10
-15
-20
I
I
I
i
i, ii
i, iii
Ru/SPS
LAP
E
GelMA 20%
GelMA 20%-Glycol Chitosan 1%
Compressive Modulus (kPa)
400
350
300
250
I
I
i
ii
Ru/SPS
LAP
F
GelMA 20%
GelMA 20%-Glycol Chitosan 1%
Swelling Ratio (%)
0
-5
-10
-15
-20
I
I
i
i
Ru/SPS
LAP
G
Beam
Cartilage-hydrogel construct
PBS bath
Testing anvil
H
GelMA 15%
GelMA 15%-Glycol Chitosan 1%
GelMA 16%
Elastic Modulus (kPa)
250
200
150
100
50
0
I
II
II
i
ii
ii
Ru/SPS
LAP

Supplementary figure S1. Compressive modulus, swelling ratio and elastic modulus of GelMA and GelMA-GC hydrogels.

10 %, 15 %, 16 % and 20 % (w v^{-1}) GelMA alone or blended with 1 % (w v^{-1}) GC was cross-linked with Ru/SPS or LAP photo-initiators at 405 nm, respectively. (A) Compressive moduli of hydrogels were measured 24 hours after polymerization using an Instron 5567 tester with a nonporous indenter and a 500 N load cell. (B) The swelling ratio was determined by weighing hydrogels directly after polymerization and after 24 hours of incubation in PBS (pH = 7.4); sample size n = 6 per group. (C) Micro-indentation test performed at 37 ºC in Cellscale micro-tester. (D) Elastic modulus calculated from the micro-indentation test by Cellscale micro-tester at 37 ºC in PBS (pH = 7.4) bath; sample size n = 4 per group. Groups that do not share a common Roman numeral are statistically different ($p < 0.05$). The comparison was conducted just inside each cross-linker group due to the differences in the cross-linking conditions and indicated by capital and lower-case numerals. GelMA (15 %, w v^{-1}) hydrogels were considered as control in each group. Error bars: Mean ± SD.

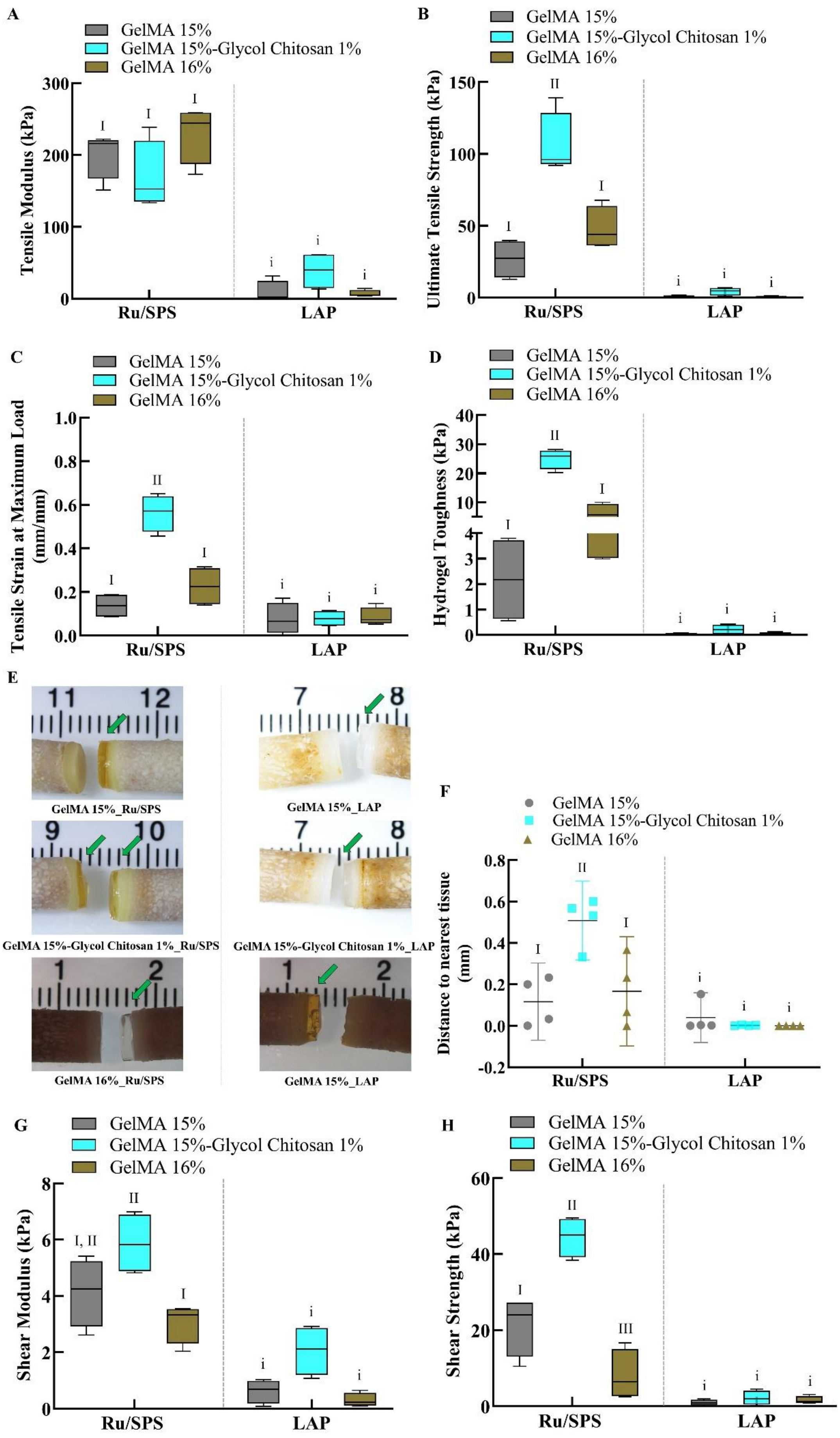

A
GelMA 15%
GelMA 15%-Glycol Chitosan 1%
GelMA 16%
Tensile Modulus (kPa)
300
200
100
0
I
I
I
i
i
i
Ru/SPS
LAP
B
GelMA 15%
GelMA 15%-Glycol Chitosan 1%
GelMA 16%
Ultimate Tensile Strength (kPa)
150
100
50
0
I
II
I
i
i
i
Ru/SPS
LAP
C
GelMA 15%
GelMA 15%-Glycol Chitosan 1%
GelMA 16%
Tensile Strain at Maximum Load (mm/mm)
1.0
0.8
0.6
0.4
0.2
0.0
I
II
I
i
i
i
Ru/SPS
LAP
D
GelMA 15%
GelMA 15%-Glycol Chitosan 1%
GelMA 16%
Hydrogel Toughness (kPa)
40
30
20
10
4
3
2
1
0
I
II
I
i
i
i
Ru/SPS
LAP
E
11
12
GelMA 15%_Ru/SPS
7
8
GelMA 15%_LAP
9
10
GelMA 15%-Glycol Chitosan 1%_Ru/SPS
7
8
GelMA 15%-Glycol Chitosan 1%_LAP
1
2
GelMA 16%_Ru/SPS
1
2
GelMA 15%_LAP
F
GelMA 15%
GelMA 15%-Glycol Chitosan 1%
GelMA 16%
Distance to nearest tissue (mm)
0.8
0.6
0.4
0.2
0.0
-0.2
I
II
I
i
i
i
Ru/SPS
LAP
G
GelMA 15%
GelMA 15%-Glycol Chitosan 1%
GelMA 16%
Shear Modulus (kPa)
8
6
4
2
0
I, II
II
I
i
i
i
Ru/SPS
LAP
H
GelMA 15%
GelMA 15%-Glycol Chitosan 1%
GelMA 16%
Shear Strength (kPa)
60
40
20
0
I
II
III
i
i
i
Ru/SPS
LAP

Supplementary figure S2. Effects of glycol chitosan on adhesive properties of GelMA and GelMA-GC hydrogels.

15 % and 16 % (w v^{-1}) GelMA alone or blended with 1 % (w v^{-1}) GC hydrogels were cross-linked with Ru/SPS and LAP photo-initiators in between the cartilage surfaces of bovine osteochondral constructs. (A) Tensile modulus, (B) ultimate tensile strength, (C) tensile strength at maximum load and (D) hydrogel toughness was measured after polymerization and after 1 hour incubation with PBS (pH = 7.4). (E) Images of the osteochondral constructs and hydrogels after adhesion tests; green arrows indicate hydrogel-cartilage integration (F) Length of hydrogel to nearest cartilage tissue was measured after adhesion tests (0 mm indicates no hydrogel on the nearest tissue). (G) Shear modulus and (H) shear strength measured in push-out test after overnight incubation in PBS at 37 ºC. Groups that do not share a common Roman numeral are statistically different ($p < 0.05$). The comparison was conducted just inside each cross-linker group, as indicated by capital and lower-case numerals. GelMA (15 %, w v^{-1}) hydrogels were considered as control in each group. Sample size n = 4 per group; error bars: Mean ± SD.

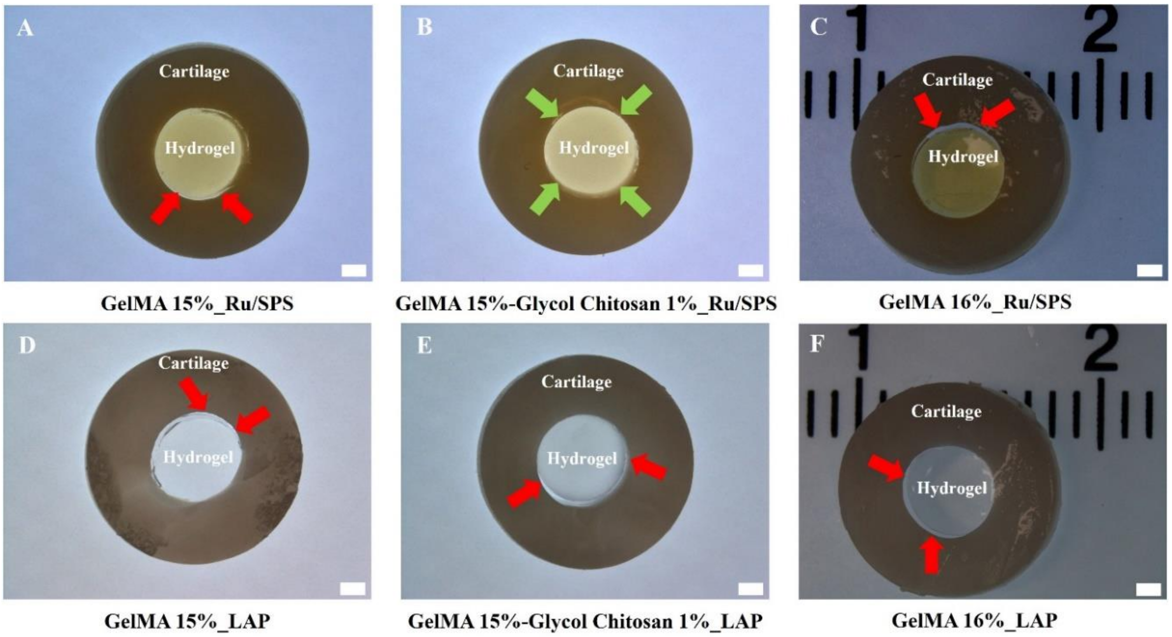

Supplementary figure S3. Stereomicroscopic images of cartilage-hydrogel construct after overnight incubation at 37 ^{o}C.

15 % and 16 % (w v^{-1}) GelMA alone or blended with 1 % (w v^{-1}) GC hydrogels were cross-linked with Ru/SPS (A - C) or LAP photo-initiators (D - F) in central defect of Bovine cartilage. Integration of hydrogels with native cartilage (robust integration marked as green arrows; weak integration marked as red arrows). Scale bar: 1000 µm.

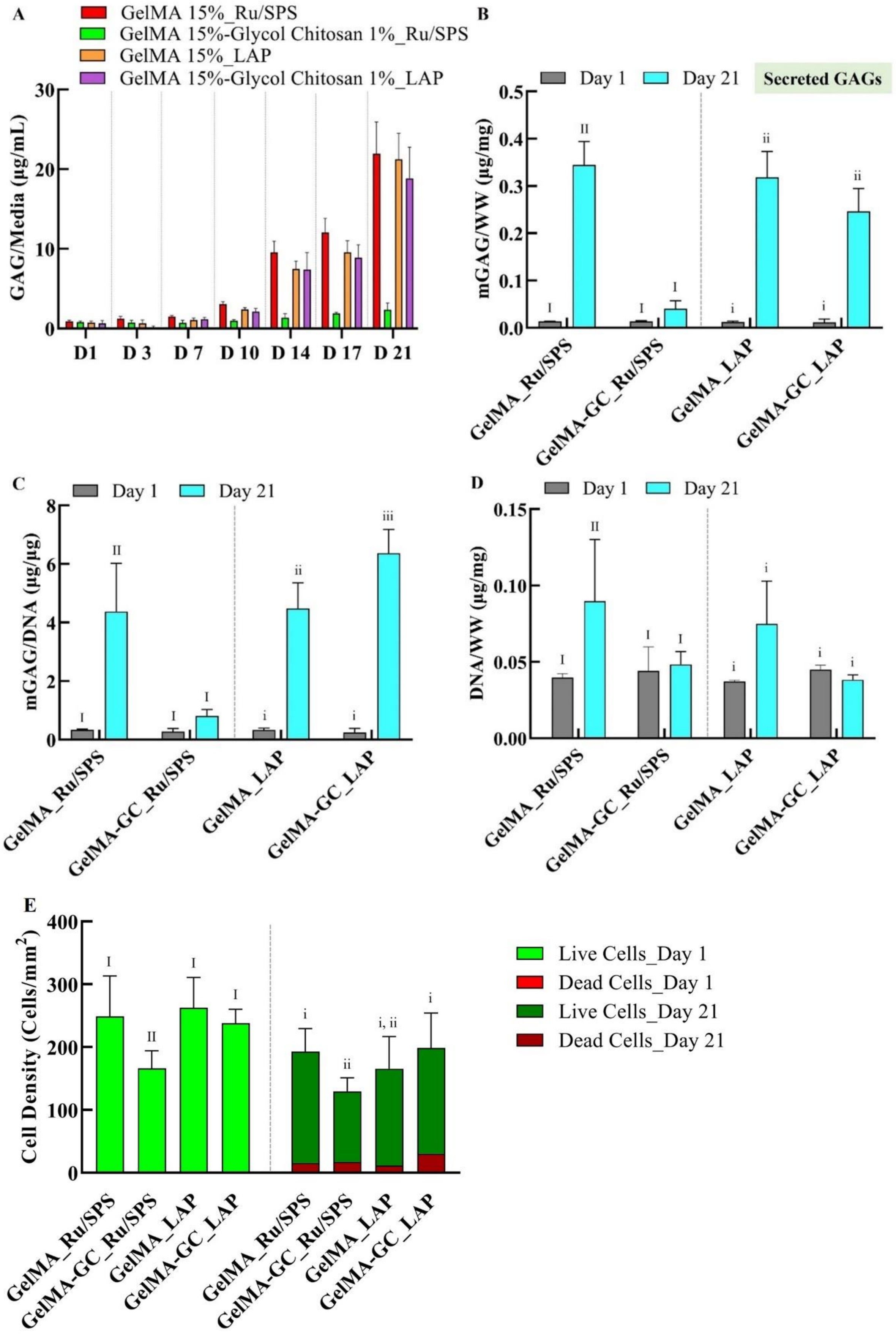

A
GelMA 15%_Ru/SPS
GelMA 15%-Glycol Chitosan 1%_Ru/SPS
GelMA 15%_LAP
GelMA 15%-Glycol Chitosan 1%_LAP
GAG/Media (µg/mL)
D1
D 3
D 7
D 10
D 14
D 17
D 21
B
Day 1
Day 21
Secreted GAGs
mGAG/WW (µg/mg)
GelMA_Ru/SPS
GelMA-GC_Ru/SPS
GelMA_LAP
GelMA-GC_LAP
C
mGAG/DNA (µg/µg)
D
DNA/WW (µg/mg)
E
Cell Density (Cells/mm2)
Live Cells_Day 1
Dead Cells_Day 1
Live Cells_Day 21
Dead Cells_Day 21

Supplementary figure S4. Biochemical properties of GelMA (15 %; w v^{-1}) alone or blended with 1 % (w v^{-1}) GC hydrogels cross-linked with Ru/SPS and LAP photo-initiators at 405 nm.

(A) Total GAGs content of media containing hydrogels at different time points (no statistical analysis was done). GAGs secreted into the media at 21 days of culture (B) normalized to hydrogel wet weight and (C) DNA content. (D) DNA content normalized to hydrogel wet weight. The comparison was conducted just inside each cross-linker group, as indicated by capital and lower-case numerals. Groups that do not share a common Roman numeral are statistically different ($p < 0.05$). GelMA (15 %, w v^{-1}) hydrogels were considered as control. Sample size n = 5 and error bars: Mean ± SD. (E) Cell density in hydrogels counted by Image J software after live/dead assay. Statistical analysis was done based on total cell density (live and dead cells/mm^2) at two different time points (day 1 and day 21) indicated by capital and lower-case numerals. Groups that do not share a common Roman numeral are statistically different ($p < 0.05$). GelMA (15 %, w v^{-1}) hydrogels were considered as control. Sample size n = 3 and error bars: Mean ± SD.

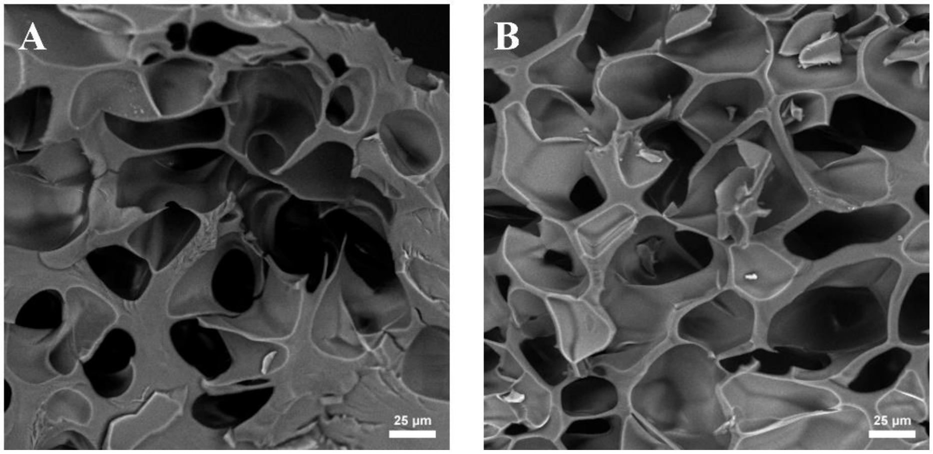

Supplementary Figure S5. Scanning electron microscopy (SEM) images depicting the internal microstructure of hydrogels (A) 15 % (w v^{-1}) GelMA and (B) 15 % (w v^{-1}) GelMA blended with 1 % (w v^{-1}) GC cross-linked by Ru/SPS photo-initiators at 405 nm. Scale bar: 25 µm.

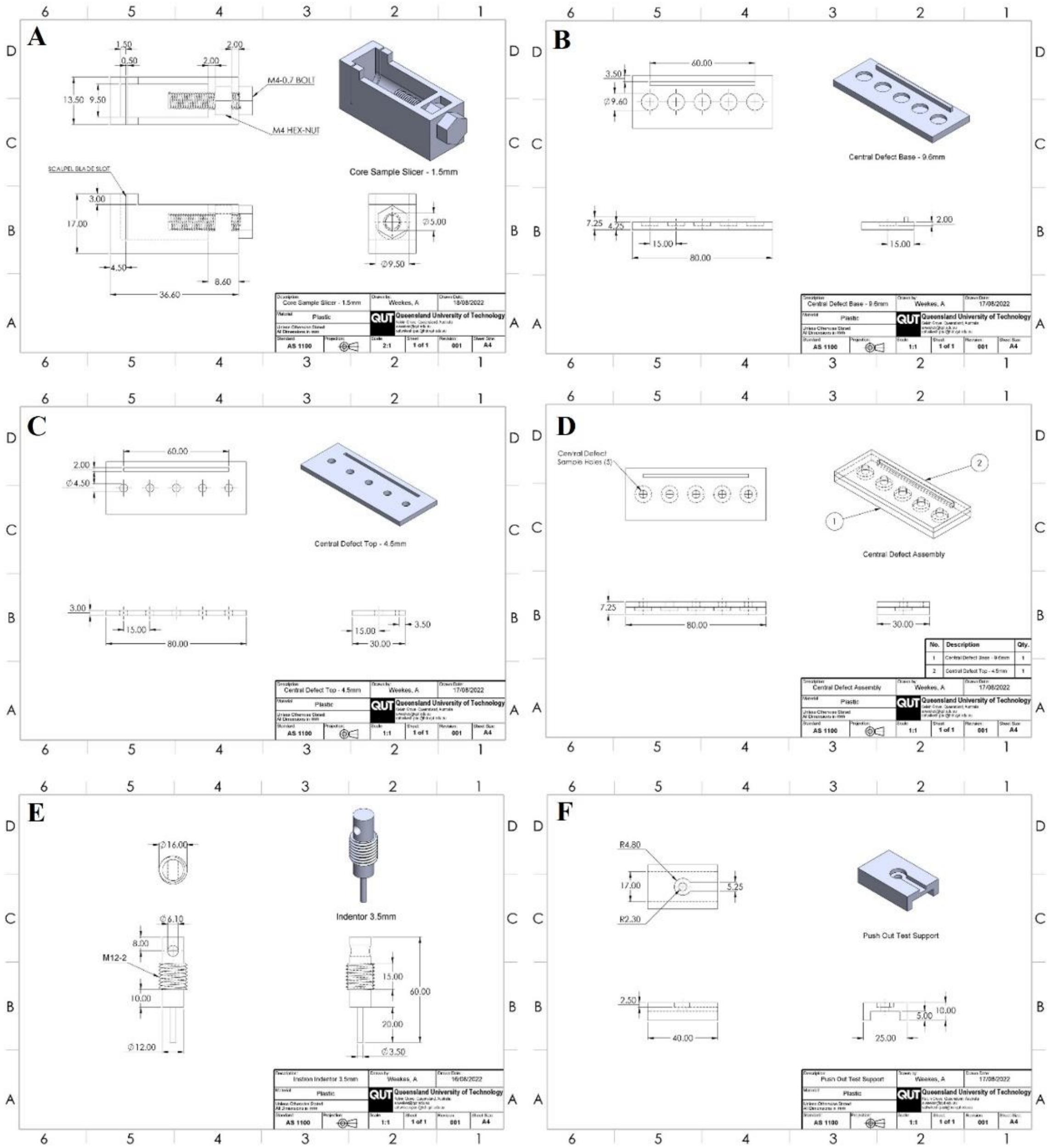

Supplementary figure S6. Technical drawing and dimensions for the custom-made plastic mould for articular cartilage slice and rings preparation.

(A) mould used for cartilage slice preparation. (B – D) mould used for creating central defect in the cartilage slice. (E) Indenter used for pushing out the hydrogel from the central defect of bovine cartilage rings, and (F) support stage used for placing the cartilage-hydrogel construct in PBS bath.
